# Loss of Slc4a1b Chloride/Bicarbonate Exchanger Function Protects Mechanosensory Hair Cells from Aminoglycoside Damage in the Zebrafish Mutant *persephone*

**DOI:** 10.1371/journal.pgen.1002971

**Published:** 2012-10-11

**Authors:** Dale W. Hailey, Brock Roberts, Kelly N. Owens, Andrew K. Stewart, Tor Linbo, Remy Pujol, Seth L. Alper, Edwin W. Rubel, David W. Raible

**Affiliations:** 1 Department of Biological Structure, University of Washington, Seattle, Washington, United States of America; 2 Virginia Merrill Bloedel Hearing Research Center, University of Washington, Seattle, Washington, United States of America; 3 Department of Otolaryngology–Head and Neck Surgery, University of Washington, Seattle, Washington, United States of America; 4 Renal Division and Molecular and Vascular Medicine Unit, Beth Israel Deaconess Medical Center, Boston, Massachusetts, United States of America; 5 INSERM Unit 583, Universite de Montpellier, Institut des Neurosciences de Montpellier, Hopital St. Eloi, Montpellier, France; Tel Aviv University, Israel

## Abstract

Mechanosensory hair cell death is a leading cause of hearing and balance disorders in the human population. Hair cells are remarkably sensitive to environmental insults such as excessive noise and exposure to some otherwise therapeutic drugs. However, individual responses to damaging agents can vary, in part due to genetic differences. We previously carried out a forward genetic screen using the zebrafish lateral line system to identify mutations that alter the response of larval hair cells to the antibiotic neomycin, one of a class of aminoglycoside compounds that cause hair cell death in humans. The *persephone* mutation confers resistance to aminoglycosides. 5 dpf homozygous *persephone* mutants are indistinguishable from wild-type siblings, but differ in their retention of lateral line hair cells upon exposure to neomycin. The mutation in *persephone* maps to the chloride/bicarbonate exchanger *slc4a1b* and introduces a single Ser-to-Phe substitution in zSlc4a1b. This mutation prevents delivery of the exchanger to the cell surface and abolishes the ability of the protein to import chloride across the plasma membrane. Loss of function of zSlc4a1b reduces hair cell death caused by exposure to the aminoglycosides neomycin, kanamycin, and gentamicin, and the chemotherapeutic drug cisplatin. Pharmacological block of anion transport with the disulfonic stilbene derivatives DIDS and SITS, or exposure to exogenous bicarbonate, also protects hair cells against damage. Both *persephone* mutant and DIDS-treated wild-type larvae show reduced uptake of labeled aminoglycosides. *persephone* mutants also show reduced FM1-43 uptake, indicating a potential impact on mechanotransduction-coupled activity in the mutant. We propose that tight regulation of the ionic environment of sensory hair cells, mediated by zSlc4a1b activity, is critical for their sensitivity to aminoglcyoside antibiotics.

## Author Summary

The majority of hearing loss in humans is caused by death of inner ear mechanosensory hair cells. The aminoglycosides, a family of antibiotics, cause permanent hearing loss and transient vestibular disorders in this fashion. If we can find ways to reduce the hair cell toxicity of these antibiotics, we will be better able to treat tuberculosis, meningitis, and infections arising from chronic conditions like cystic fibrosis. Our lab uses zebrafish as a tool to identify ways to block hair cell death. Zebrafish have hair cells on their surface that are killed by aminoglycosides. We generated a pool of mutants and identified fish that retain hair cells when exposed to aminoglycosides. Here we describe why one of these mutants, *persephone*, protects hair cells from aminoglycosides. *persephone* has a mutation in a chloride/bicarbonate exchanger. This mutant protein fails to move chloride across cell membranes. Loss of the protein's activity decreases aminoglycoside entry into hair cells. We speculate that the exchanger mutated in *persephone* may be a critical regulator of ionic conditions that promote entry of aminoglycosides into hair cells. The *persephone* mutant furthermore suggests that transiently altering the ionic environment of hair cells may be a useful approach to protect hair cells from aminoglycoside exposure.

## Introduction

The aminoglycoside antibiotics are structurally related compounds that include familiar drugs such as neomycin, kanamycin, gentamicin, and streptomycin. Aminoglycosides are routinely used to treat gram-negative bacterial infections, and some gram-positive and mycobacterial infections (e.g. *M. tuberculosis*). The first clinical use of aminoglycosides, streptomycin in 1944, was followed shortly by reports of ototoxicity and nephrotoxicity [1]. Hearing loss from aminoglycoside exposure occurs primarily through death of the mechanosensory hair cells of the inner ear–the cells that first convert fluid pressure changes generated by sound into synaptic activity. Nephrotoxicity arises from death of proximal kidney tubule epithelia [2]. Additionally, aminoglycoside-induced death of hair cells in the vestibular system causes balance disorders [3]. Although numerous natural and synthetic aminoglycosides have been identified, all are associated with significant risk of hearing loss [4]–[7]. Despite these contraindications, and despite the absence of a pharmaceutical intervention to prevent their ototoxicity, aminoglycoside antibiotics remain in use today as effective therapeutics against life-threatening infections. If aminoglycoside toxicity to hair cells and renal proximal tubule cells could be blocked or attenuated, the resultant increase in the therapeutic window would greatly improve the efficacy and safety of aminoglycoside therapy [8].

To identify genetic factors that control hair cell sensitivity to aminoglycoside exposure, and to find candidate targets for drugs that could block the ototoxic effects of aminoglycosides, we developed a genetic screen for modulators of aminoglycoside hair cell toxicity in the zebrafish [9]. The location of mammalian inner ear hair cells within the temporal bone poses a challenge to large-scale screening. Zebrafish (*Danio rerio*), like most aquatic vertebrates, have hair cells on their surface in structures called neuromasts [10]–[12], and therefore provide a convenient model in which to study hair cell death. These external hair cells are part of the lateral line, a sensory system used by fish and amphibians to detect movement in surrounding water [13], [14]. The superficial hair cells of the lateral line are structurally and functionally similar to mammalian hair cells and are easy to observe and monitor [15]. We evaluated lateral line hair cell sensitivity to aminoglycoside exposure in an F3 forward genetic screen and identified zebrafish mutants that retain hair cells when exposed to neomycin [9].

In this study we show that the zebrafish mutant *persephone* protects hair cells from aminoglycoside-induced hair cell death due to a mutation in *slc4a1b*, encoding an anion exchanger of the solute carrier 4 (Slc4) family of bicarbonate transporters. We show that the missense mutation in *slc4a1b* results in failure of the protein to traffic to the plasma membrane and in consequent loss of exchange activity. We find that increasing bath bicarbonate concentration or pharmacological inhibition of anion exchanger activity protects wildtype hair cells from aminoglycoside exposure. Lastly, we show that the protection conferred by loss of the bicarbonate/chloride exchanger likely reflects reduced aminoglycoside entry into hair cells, and that this is potentially influenced by a decrease in mechanotransduction activity in the mutant.

## Results

### *persephone* confers resistance to neomycin damage m

**Figure 1:**
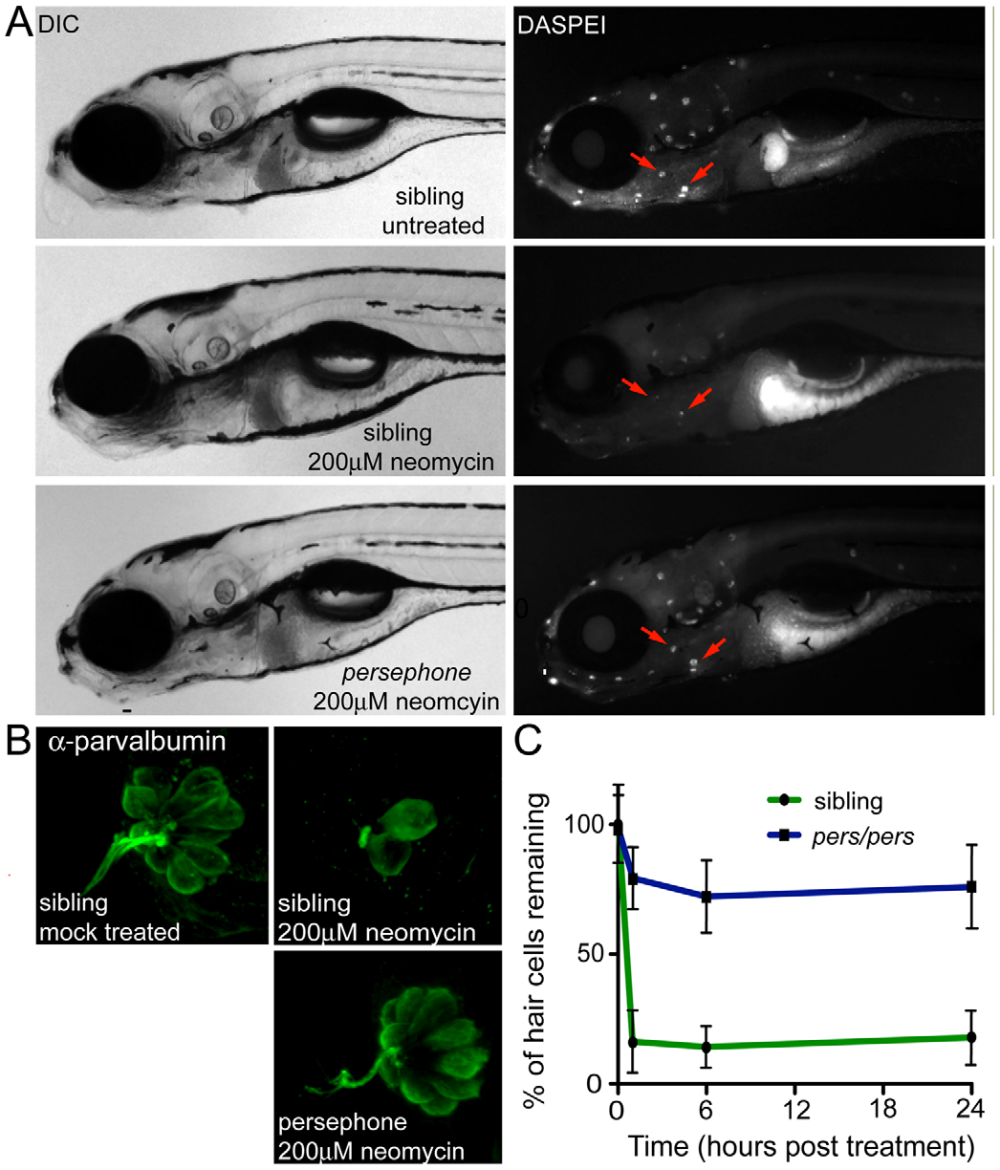
Phenotype of the *persephone* mutant. (A) Hair cell protection in homozygous *persephone* mutants. 5 dpf zebrafish (progeny of a heterozygous incross) were treated with or without 200 µM neomycin for 30 min, and then rinsed. After 1 hr recovery in fresh embryo media, hair cells were labeled with the vital dye DASPEI. Left panels are differential interference contrast (DIC) images and right panels are corresponding fluorescent images. Top, wildtype sibling (+/+), mock-treated, Middle, wildtype sibling (+/+), treated with 200 µM neomycin, and Bottom, *persephone* homozygote treated with 200 µM neomycin. Red arrows indicate examples of neuromasts present in the untreated wildtype and treated *persephone* larvae. Mutant larvae show dramatic retention of hair cells relative to their siblings. (B) Parvalbumin antibody staining of hair cells in representative fish from an in-cross of *persephone* heterozygotes. Wildtype siblings show loss of hair cells when treated with 200 µM neomycin. Homozygous *persephone* mutants show a dramatic retention of parvalbumin-stained hair cells following neomycin treatment, consistent with DASPEI hair cell staining results. (C) Protection observed in *persephone* is not due to a delay in neomycin-induced hair cell death. *persephone* mutants (blue line) exposed to neomycin for 1 hr, rinsed and maintained for 6 or 24 hr in fresh EM, and then assessed for hair cell death, do not show significantly greater hair cell death than those assayed after 1 hr. Hair cell death is not delayed in *persephone*. Wildtype siblings treated in parallel (green line) are shown for comparison. (n = 10 fish, 10 neuromasts per fish; Error bars: S.D.; p value<0.001).

The *persephone* mutant was identified in a genetic screen by its ability to retain lateral line mechanosensory hair cells following a 30 min exposure to 200 µM neomycin [9]. Hair cells of 5 days post-fertilization (dpf) wildtype zebrafish show robust labeling with the vital dye DASPEI, appearing as bright puncta on the larval surface (Figure 1A, top panel) [16]. Treatment of wildtype zebrafish larvae with neomycin kills >85% of lateral line hair cells, resulting in loss of DASPEI staining (Figure 1A, middle panel). In contrast, homozygous *persephone (pers*) mutant larvae retain most of their lateral line hair cells after neomycin treatment (Figure 1A, bottom panel). Notably, *persephone* mutants are otherwise indistinguishable from their wildtype siblings; they show normal swim bladder inflation, normal jaw and otolith development, normal pigmentation, etc. (see DIC panels). We confirmed resistance of *pers* hair cells to neomycin treatment by staining them with an antibody to the hair cell marker parvalbumin [17], [18]. Using this assay, hair cell protection in neomycin-treated *persephone* fish (Figure 1B) was comparable to that found in untreated *pers* mutants assayed with DASPEI staining.

We next tested whether the resistance of *pers* mutant hair cells to neomycin exposure is simply the consequence of a delay in hair cell death. We exposed animals to neomycin as before (30 min at 200 µM), then washed the larvae in fresh embryo media, and maintained them in the absence of aminoglycosides for 24 hrs, a period of time long enough to observe any additional hair cell loss but before any substantial hair cell regeneration takes place [19]. No additional cell death was noted, suggesting that hair cell protection is due to stable inhibition of toxicity in *persephone*, not to a delay in the lethal response to neomycin (Figure 1C).

### *persephone* carries a lesion in the bicarbonate/chloride exchanger Slc4a1b

**Figure 2:**
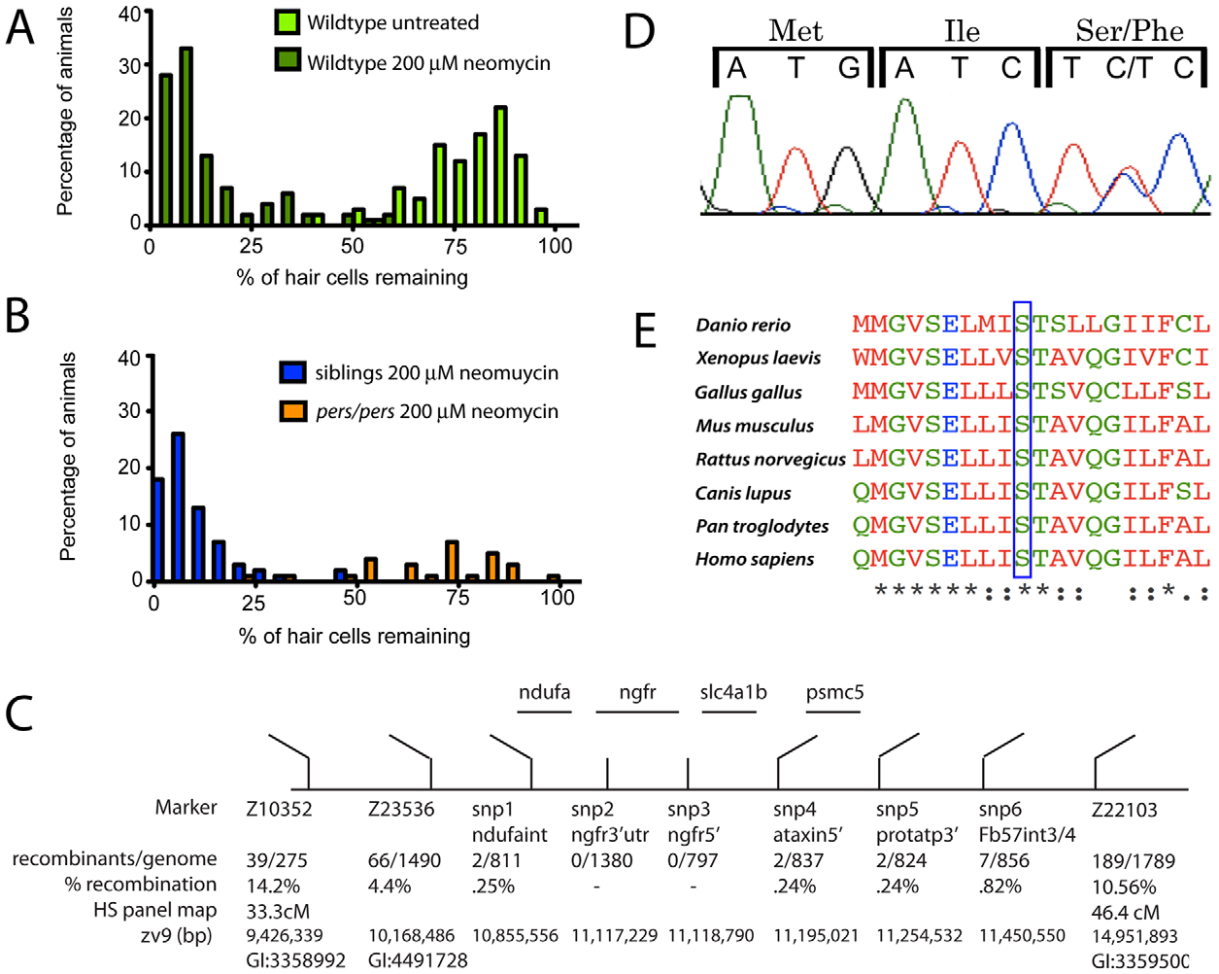
Mapping the *persephone* mutant. (A) Histograms showing the distribution of hair cell labeling in wildtype fish. 40 individual wildtype fish were mock-treated (light green bars) or treated with 200 µM neomycin for 30 min (dark green bars). Mock-treated wildtype larvae show a distribution centered near 85%. The distribution is dramatically reduced in larvae exposed to neomycin (mean centered near 10%). (B) Histogram showing distribution of hair cell retention in progeny from an in-cross of *persephone* heterozygotes. Individual larvae were assayed for hair cell staining, and then genotyped (see Materials and Methods). Orange bars show binned hair cell staining of homozygous *persephone* mutants; blue bars show binned hair cell staining of wildtype siblings (both *pers/+* and *+/+*). The distribution of hair cell retention in *persephone* homozygotes is dramatically shifted with a mean broadly centered around 80%. In contrast, heterozygous and homozygous wildtype siblings do not show protection from neomycin exposure; their mean distribution is centered around 5%. (C) Schematic diagram of the region of zebrafish chromosome 12 that cosegregates with the *persephone* mutation. Genetic markers used for fine mapping are indicated with the number and % of recombinants observed. Positions on the heat shock (HS) genetic map and the physical map are from the Zv9 genomic assembly. (D) Sequencing chromatogram of a *persephone* heterozygote showing the mutated codon and the two preceding codons. The C→T mutation in *persephone* converts the wildtype Ser to Phe at amino acid 298 in the mutant allele. (E) Alignment of zslc4a1b homologs shows evolutionary conservation of Ser 298 mutated in the *persephone* mutant. The indicated serine residue is invariant across a diverse range of taxa; see also Figure S1.

The strong degree of hair cell protection in the *persephone* mutants makes it possible to separate mutants from their siblings. The hair cells of wildtype larvae are very sensitive to neomycin exposure (Figure 2A). In a cross of *persephone* mutant heterozygous carriers, hair cell survival in offspring following exposure to 200 µM neomycin and subsequent DASPEI staining shows a bimodal distribution (Figure 2B). We initially used this distribution to designate an individual as wildtype (< = midpoint of lower peak) or mutant (> = midpoint of the higher peak). For genetic mapping, individual fish were phenotyped, and DNA was isolated from 80 individuals to create 2 mutant and 2 wildtype pools for bulk segregant analysis [20]. Initial linkage mapping was confirmed using DNA from individual mutant and wildtype animals. A region of chromosome 12 bound by the markers Z23536 and Z22103 co-segregated with the *persephone* mutation, and this region was refined using SNP markers to identify the genetic interval schematized in Figure 2C. We sequenced the four genes present in this linkage region using genomic DNA. In three of these genes there were no coding changes relative to the reference genome other than those also present in wildtype populations. However, we identified a cytosine-to-thymine point mutation in the formerly uncharacterized ORF LOC561787, now annotated as *slc4a1b* [21]. This point mutation converts Serine 298 to Phenylalanine (Figure 2D). Clustal analysis of *slc4a1b* homologs shows that this serine is invariant across diverse taxa (Figure 2E, also Figure S1).

The S298F mutation co-segregates with hair cell protection; all mutants carry the serine to phenylalanine missense mutation. The mutation is fully recessive; S298F heterozygotes exhibit normal neomycin-sensitivity, and are morphologically indistinguishable from wildtype individuals. There is no lethality associated with *persephone* among 5–9 dpf zebrafish. We have observed reduced viability of *persephone* homozygotes during juvenile development, although some individuals can survive to adulthood and are fertile. The mutation does not appear to alter mRNA stability. *In situ* analysis of zSlc4a1b expression using a full-length probe showed high mRNA expression in ionocytes in the skin, as well as weak expression throughout the embryo, as previously reported by Lee et al [21]. *pers* mutants exhibit the same expression pattern (data not shown).

### *persephone* mutants protect hair cells from multiple hair cell toxins

**Figure 3:**
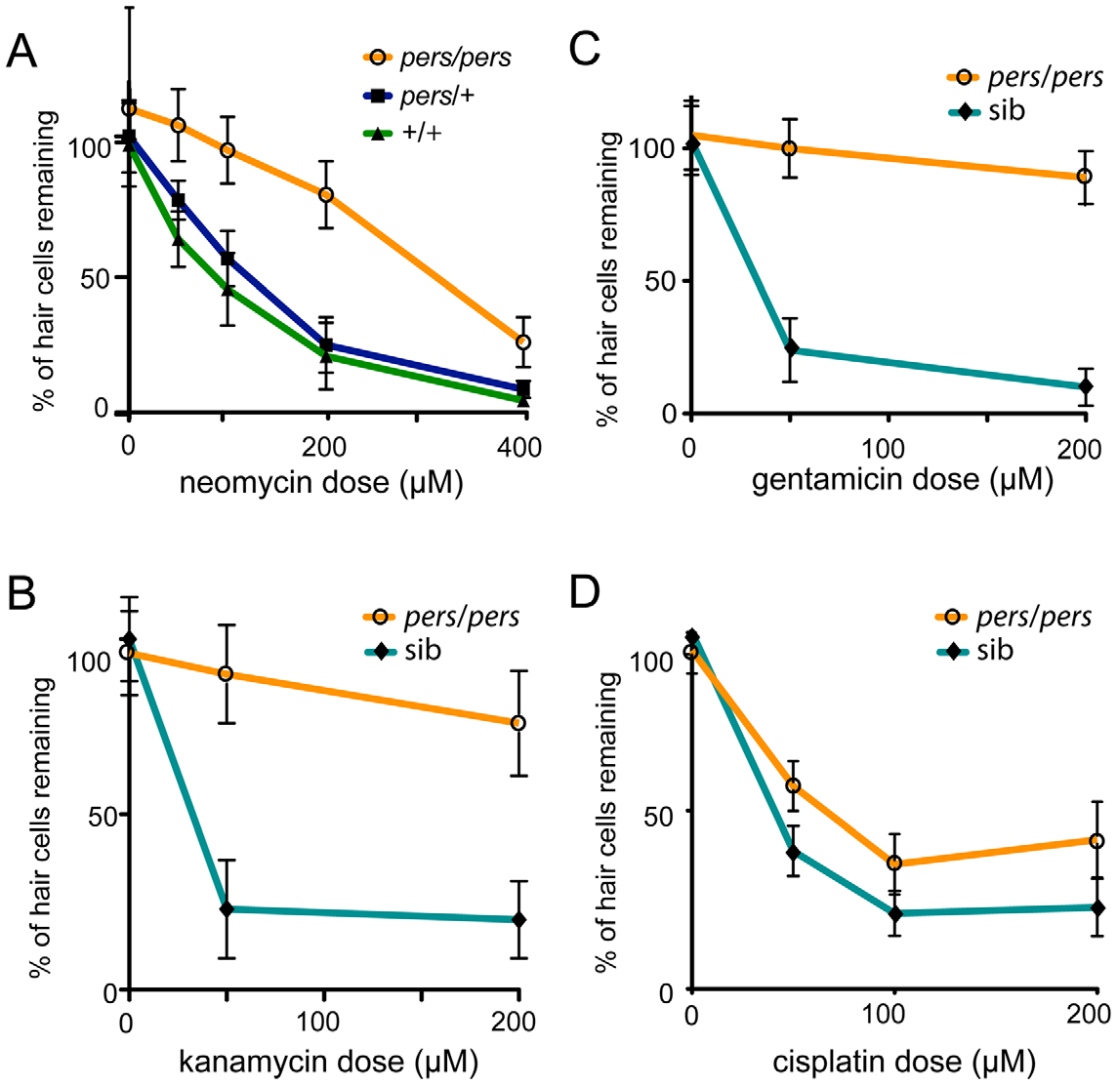
Protection of hair cells in the *persephone* mutant. (A) Neomycin dose response curve for progeny from a *persephone* incross. Homozygous *persephone* mutants (orange line) show dramatic protection at all tested neomycin concentrations (50, 100, 200 and 400 µM) as compared to wildtype (green line) and heterozygous siblings (blue line) that show no protection. (n≥10 fish per group, 10 neuromasts per fish. Error bars: S.D.; ANOVA p value<0.0001) (B) *persephone* protects hair cells from loss induced by the aminoglycoside kanamycin. Larvae were maintained in embryo media with kanamycin (0, 50, and 200 µM) for 24 hr prior to assaying hair cell death. Mutants (orange line) are protected as compared to the combined wildtype and heterozygous siblings (teal line). (n≥10 fish per group, 10 neuromasts per fish. Error bars: S.D.; ANOVA p value<0.0001) (C) *persephone* protects hair cells from loss induced by the aminoglycoside gentamicin. Larvae were maintained in embryo media with gentamicin (0, 50, and 200 µM) for 6 hr prior to assaying hair cell death. Mutants (orange line) are protected as compared to the combined wildtype and heterozygous siblings (teal line). (n≥10 fish per group, 10 neuromasts per fish. Error bars: S.D.; ANOVA p value<0.0001) (D) *persephone* protects against the hair cell toxin cisplatin. Larvae were exposed for 24 hr to cisplatin (50, 100 or 200 µM) and assayed for hair cell survival by hair cell counts prior to genotyping. Homozygous *persephone* mutants (orange line) show modest but significant protection compared to wildtype siblings (teal line) (n≥10 fish per group, 3 neuromasts per fish. Error bars: S.D.; ANOVA p value<0.0001.).

We next tested the response of *persephone* mutants to a panel of hair cell toxins over a range of concentrations, using DASPEI staining or hair cell counts and subsequent genotyping of individual larvae. Heterozygote *pers* siblings and wildtype larvae showed equivalent degrees of hair cell death when exposed to neomycin (Figure 3A). In contrast, homozygous *persephone* larvae showed hair cell protection over neomycin doses ranging from 50 to 400 µM. At 400 µM, hair cell death in *pers* larvae is substantial, but hair cell survival in mutants is still >5 fold higher than that seen in wildtype siblings (Figure 3A). *pers* larvae exposed to the aminoglycosides kanamycin (Figure 3B) and gentamicin (Figure 3C) also showed resistance compared to wildtype siblings. The *persephone* mutation also protected hair cells exposed to the chemotherapeutic drug cisplatin, a well-described hair cell toxin [22], [23] (Figure 3D). The degree of protection from a 24 hour cisplatin exposure is markedly lower; we were only able to observe this using hair cell counts of antibody-stained tissue–a method more discriminating than DASPEI staining. However, protection from cisplatin is significant. These findings suggest that *persephone* mutants likely either affect an early stage in hair cell sensitivity common to aminoglycosides and cisplatin, or a very late stage in death commitment.

### *persephone* protects from the earliest intracellular changes induced by aminoglycoside exposure

**Figure 4:**
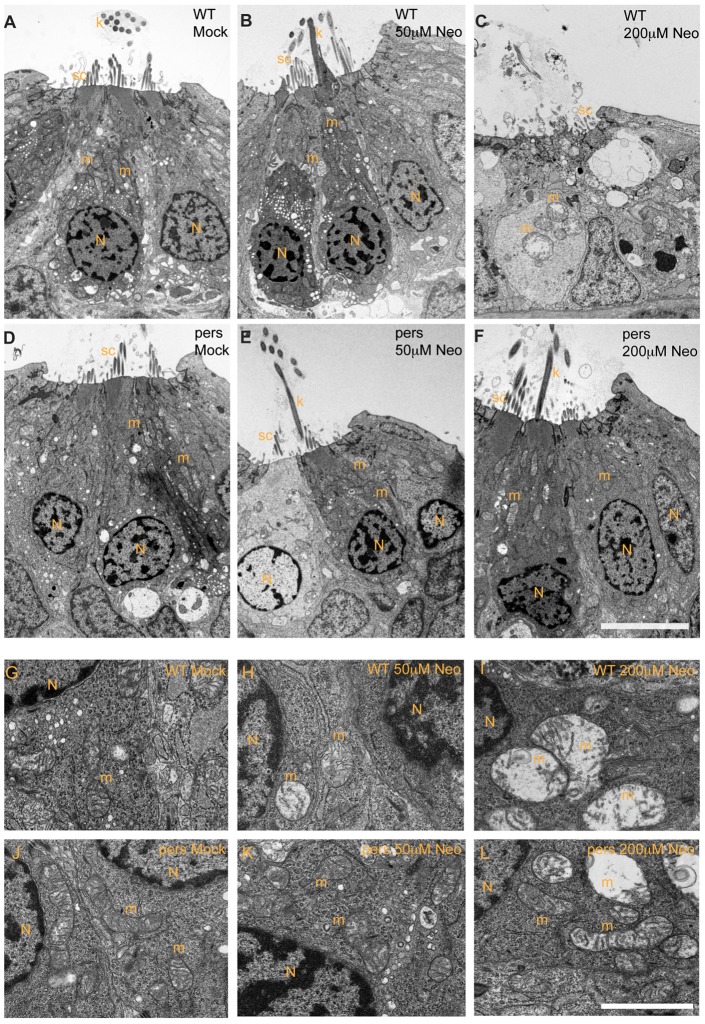
*persephone* hair cells have normal morphology and show only modest ultrastructural changes when treated with neomycin. Zebrafish (5 dpf) treated with or without neomycin were euthanized, tails were removed for genotyping, and heads were fixed for transmission electron microscopy. Images of transverse sections through neuromasts are shown. (N: hair cell nuclei, m:mitochondria, sc: support cell, k: kinocilia. Top panels A–C and bottom panels G–I show hair cells from wildtype siblings. Top panels D–F and bottom panels J–L show hair cells from *persephone* homozygotes. Panels were either mock-treated or exposed to 50 µM or 200 µM neomycin. Hair cells of mock-treated wildtype siblings and *persephone* mutants show organized stereocilia, a large central nucleus, and normal mitochondrial morphology. There are no readily apparent differences between the wildtype siblings and *persephone* mutants. Hair cells of *persephone mutants* treated with 50 µM neomycin show either mild mitochondrial swelling (E,K) or are indistinguishable from mock-treated wildtype siblings. In comparison wildtype siblings (B,H) show swollen mitochondria and fused stereocilia. Most neuromasts from wildtype siblings treated with 200 µM neomycin lack hair cells, and remaining hair cells exhibit severe damage including condensed nuclei and severely swollen mitochondria or signs of cytolysis (C,I). Hair cells of *persephone* mutants treated with 200 µM neomycin show minimal damage. Stereocilia are typically oraganized and intact; some but not all hair cells show modestly swollen mitochondria (F,L). See Table 1 for quantification of neomycin-induced damage across genotypes. Top scale bar = 5 µm; Bottom scale bar = 1 µm.

We previously showed that mitochondrial swelling is one of the first morphological changes seen in lateral line hair cells after neomycin treatment, and is the prevalent change with low (25 µM) neomycin exposure [24]. Treatment with high aminoglycoside concentration (200 µM) for 15 min to 1 hr results in more dramatic changes including mitochondrial dysmorphology, evulsion of the cuticular plate, stereocilia fusion, nuclear condensation/pyknosis, and cellular condensation. To determine whether *persephone* mutant larvae display the changes in ultrastructure after aminoglycoside exposure seen in wildtype larvae, we prepared *persephone* mutants and their wildtype siblings for TEM analysis, saving a portion of tissue for genotyping. Hair cells of mock-treated *persephone* homozygotes were indistinguishable from wildtype siblings (compare Figure 4A, 4D and 4G, 4J). Hair cells in wildtype animals treated with 200 µM neomycin for 1 hr displayed damage similar to that previously described. Most neuromasts showed extensive loss of hair cells; remaining hair cells showed dramatic morphological changes including severely swollen mitochondria and disrupted stereocilia structure and organization (Figure 4C, 4I). In contrast, *persephone* mutants treated with 200 µM neomycin for 1 hr typically exhibited modest changes in mitochondrial morphology. Many mitochondria appeared normal. Kinocilia and stereocilia also largely showed normal structure and organization (Figure 4F, 4L and Table 1). The level of damage in *persephone* mutants treated with 200 µM neomcycin was roughly comparable to wildtype zebrafish treated with 25–50 µM neomycin (Figure 4B, 4H). These observations indicate that *persephone* attenuates aminoglycoside responses prior to their induction of detectable changes in hair cell ultrastructure.

**Table 1:** Ultrastructural damage is strongly reduced in *persephone* homozygotes versus wild-type siblings after neomycin exposure.

Treatment	Genotype	A–B	C	D	E
mock	*pers*/*pers*	4	0	0	2
	*pers*/+	3	0	0	2
	+/+	4	2	0	4
50 µM Neo	*pers/pers*	7	1	0	2
	*pers*/+	3	2	0	2
	+/+	2	0	0	2
200 µM Neo	*pers/pers*	5	2	1	2
	*pers*/+	0	0	3	2
	+/+	1	0	5	4

n = number of fish in each category.

Damage scale:

A = All hair cells of the neuromast appear normal.

B = Slight mitochondrial swelling (modest within range of mock-treated controls).

C = One or more hair cells exhibit some mitochondrial swelling. One or more hair cells also exhibit some nuclear condensation.

D = Many hair cells show severe damage, including distended mitochondria, highly pycnotic nuclei, or condensation of the entire cell.

E = Hair cells are absent, or all hair cells show severe damage, including severely distended mitochondria, highly pycnotic nuclei, or condensation of the entire cell.

### Slc4a1b(S298F) fails to localize to plasma membrane or permit ion transport

**Figure 5:**
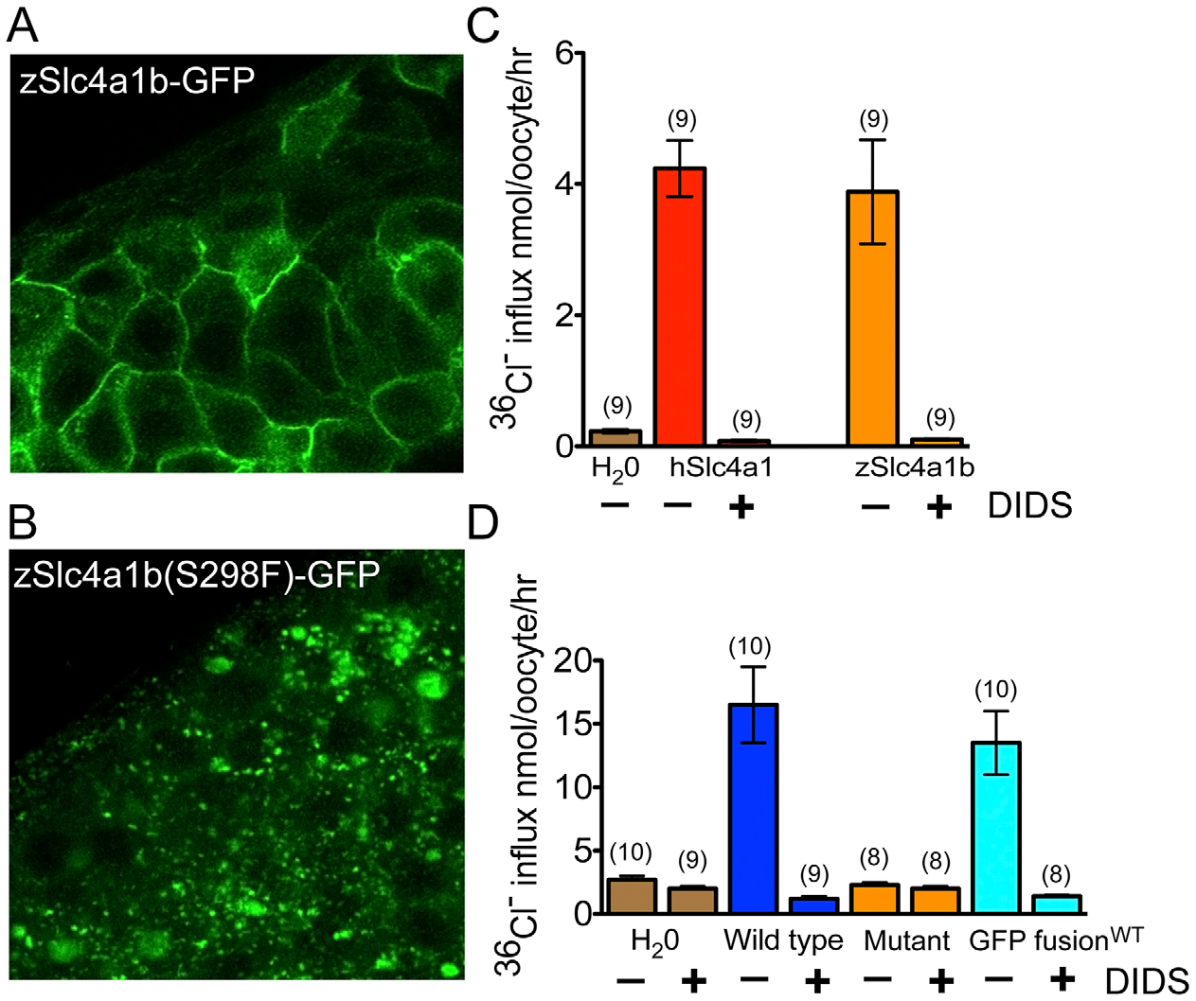
Mutant Slc4a1b is mislocalized and fails to transport chloride. (A) Confocal images of zebrafish embryos transiently expressing wildtype Slc4a1b–GFP. mRNA was injected into one cell stage embryos. Cells along the anterior dorsal aspect of embryos were imaged at 48 hpf. Wildtype protein is localized to the plasma membrane. B) Confocal images of zebrafish embryos expressing mutant Slc4a1b(S298F)-GFP. Slc4(S298F)-GFP is present in intracellular vesicles, with complete loss of plasma membrane localization. (C) Chloride influx into *Xenopus laevis* oocytes expressing either zebrafish Slc4a1b (zSlc4a1b) or the closest human homolog, SLC4A1 (hSlc4a1). Zebrafish Slc4a1b transports radiolabeled chloride across oocyte membranes as efficiently as its nearest human homolog. This activity is blocked by DIDS (200 µM), an inhibitor of Slc4 family-mediated anion exchangers. (D) Comparison of chloride influx into *Xenopus laevis* oocytes expressing wildtype or mutant (S298F) zSlc4a1b, or wildtype zSlc4a1b-GFP. zSlc4a1(S298F), the *persephone* gene product, exhibits complete loss of chloride influx activity. Notably, GFP-tagged wildtype slc4a1b exhibits no significant reduction of chloride transport activity. (n indicated above each sample; Error bars = SEM).

Slc4a1b belongs to the Na^+^-independent, electroneutral chloride/bicarbonate subfamily of Slc4 anion transporters. The most extensively characterized of these, human AE1 (human Slc4A1), is a closely related ortholog of zebrafish Slc4a1b [21]. Its role in the exchange of bicarbonate for chloride across the basolateral membranes of cells in collecting duct Type A intercalated cells of mammalian kidney has been extensively studied [25], [26]. The *persephone* mutation we identified as *D. rerio* Slc4a1b S298F corresponds to residue human AE1 S443, located in the middle of the predicted second transmembrane domain. Human AE1 S443 is flanked on one side by the Eosin-5′-maleimide binding site at K430 in the preceding extracellular loop, and on the other by the PCMBS binding site C479 located at the extracellular end of the third transmembrane span. The distal renal tubular acidosis (dRTA) mutation C479W (Band 3 Edmonton) disrupts AE1 trafficking in renal epithelial cells [27], [28]. We speculated that the S298F mutation in the zebrafish Slc4a1b protein might interfere with proper trafficking through the secretory pathway and/or delivery to the plasma membrane. To test this idea, we fused green fluorescent protein (GFP) to wildtype and mutant forms of Slc4a1b at the carboxy-terminus, and evaluated the localization of these chimeric proteins in zebrafish embryos. Injected mRNA expression for both the wildtype and mutant forms was broadly seen throughout 2 dpf embryos. In all expressing cells, wildtype Slc4a1b-GFP readily trafficked to the cell surface and strongly labeled the plasma membrane (Figure 5A). The mutant form Slc4a1b(S298F)-GFP showed a strikingly different localization. The protein failed to reach the cell surface, and instead appeared in puncta throughout the cell (Figure 5B). The distribution pattern of either wildtype or mutant protein was unchanged across all tissues examined. Based on these observations, we predicted that disruption of trafficking of the anion exchanger to the cell surface might disrupt its exchange activity at the cell surface.

Structural similarities with its orthologs suggest that zebrafish Slc4a1b carries out influx of chloride and efflux of bicarbonate. We therefore asked whether we could detect zebrafish Slc4a1b-mediated import of chloride. Chloride transport across the plasma membrane can be assayed directly by expressing transporters in *Xenopus laevis* oocytes and measuring movement of radiolabeled chloride [29]. We used this system to evaluate whether chloride transport is mediated by zebrafish Slc4a1b, and whether this activity is compromised in the S298F mutant protein. WT and mutant cRNAs were injected into *Xenopus* oocytes. Oocytes were maintained for 3 days to allow protein expression and surface delivery. The oocytes were then assayed for chloride transport. Oocytes exogenously expressing either human Slc4A1 (used as a positive control) or wildtype zebrafish Slc4a1b exhibited nearly 10-fold elevation of chloride uptake above background (Figure 5C). Notably, transport by zebrafish Slc4a1b and by human SLC4A1 was abolished by treatment with the disulfonic stilbene DIDS–an inhibitor of Slc4-mediated solute transport. Zebrafish Slc4a1b also mediated extracellular Cl^−^-dependent ^36^Cl^−^ efflux that was sensitive to inhibition by DIDS (not shown), consistent with a transport mechanism of anion exchange. However, Zebrafish Slc4a1b-mediated uptake of ^36^Cl^−^ influx was unaffected by 200 µM gentamicin (not shown).

The failure of mutant Slc4a1b to localize at the cell surface was consistent with its inability to facilitate chloride uptake into oocytes (Figure 5D). Notably, the fusion of Slc4a1b with GFP also showed robust chloride transport activity at levels comparable to that of the untagged polypeptide, suggesting that the GFP fusion does not compromise the structure of the exchanger (Figure 5D). Zebrafish Slc4a1b-GFP therefore likely recapitulates trafficking of the wildtype protein. The aberrant intracellular localization of the mutant Slc4a1b-GFP, and the inability of the untagged mutant form to transport chloride are both consistent with the coding change in *persephone* acting as an Slc4a1b loss-of-function mutation due to a trafficking failure.

### Inhibition of Slc4 transporter activity protects hair cells from aminoglycosides

**Figure 6:**
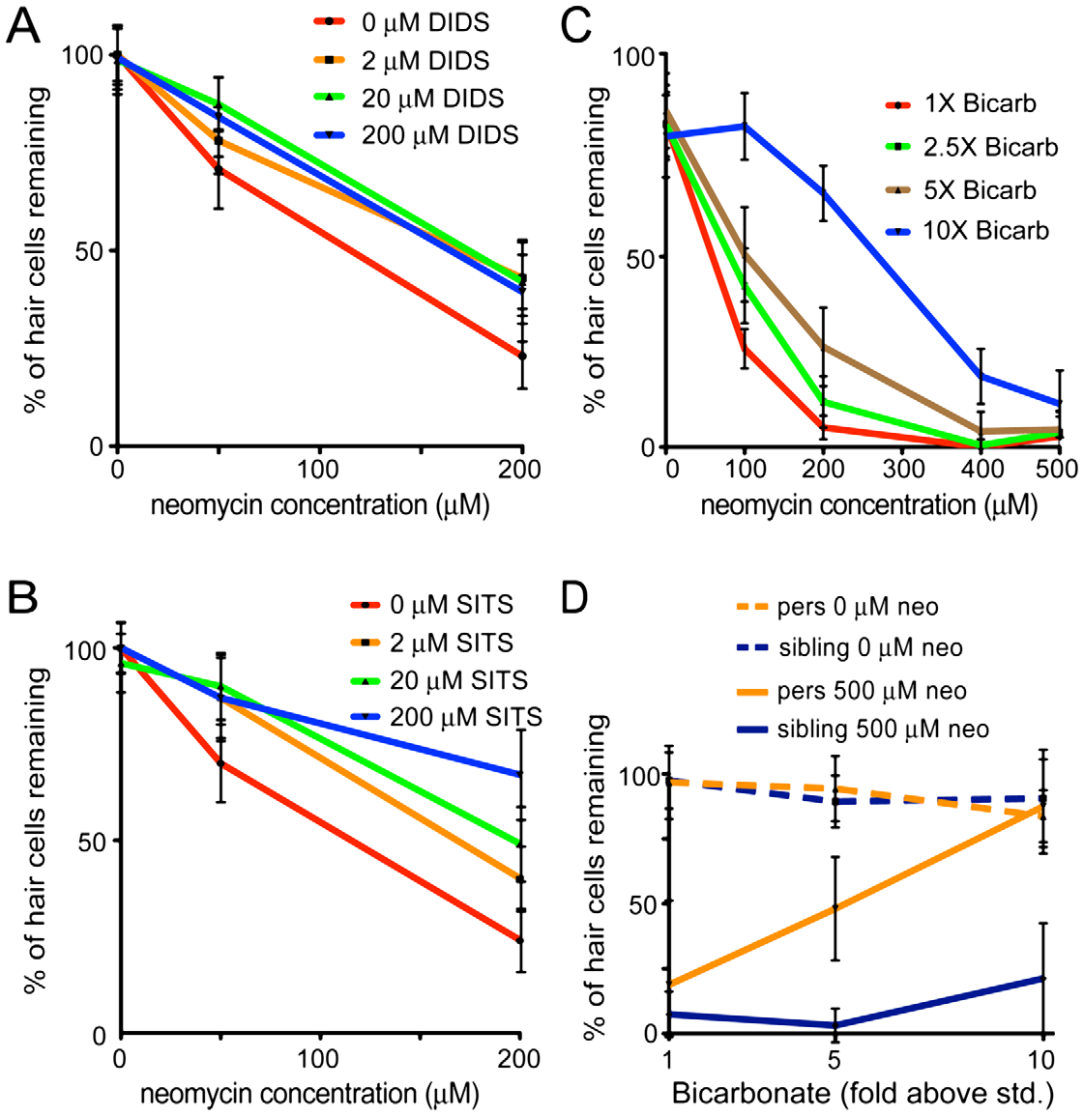
Protection of hair cells by zSlc4a1b inhibitors and substrates. (A) Treatment with the Slc4 inhibitor DIDS protects hair cells from exposure to neomycin. 5 dpf zebrafish were pretreated with DIDS for 1 hr; then co-treated with DIDS and neomycin for 1 hr. Treatment with DIDS resulted in modest but significant reduction in hair cell death. (n≥10 larvae per group, 3 neuromasts per larvae. Error bars: S.D.; ANOVA p value indicating significance of DIDS to protection: <0.001) (B) Treatment with the Slc4 inhibitor SITS protects hair cells from neomycin toxicity. SITS protects hair cells in a concentration-dependent manner against hair cell loss caused by exposure to 200 µM neomycin. (n≥10 fish per group, 3 neuromasts per fish. Error bars: S.D.; ANOVA p value indicating significance of SITS to protection: <0.0001) (C) Increased bicarbonate in the embryo media protects hair cells from exposure to neomycin. 5 dpf zebrafish were exposed for 1 hr to embryo media with a 10 fold range of bicarbonate concentrations (0.714 mM to 7 mM). Neomycin was then added at the indicated concentrations for 30 min. Larvae were rinsed three times in fresh media, and allowed to recover for 1 hr. Bicarbonate protects hair cells in a concentration-dependent manner. (n≥10 fish per group, 3 neuromasts per fish. Error bars: S.D.; ANOVA p value indicating significance of bicarbonate to protection: <0.001). See also Figure S2. (D) *persephone* mutants show dramatically increased bicarbonate-mediated hair cell protection relative to heterozygous and homozygous wildtype siblings. Progeny of an in-cross of *persephone* heterozygotes (5 dpf) were exposed for 1 hr to embryo media with bicarbonate added at the indicated concentrations, and then treated an additional 1 hr with or without 500 µM neomycin. Fish were allowed to recover for 1 hr, then were assayed individually for hair cell survival prior to euthanasia and genotyping by dCAPS. Whereas 10× [bicarbonate] only modestly protected wildtype and heterozygous siblings (solid blue line), *persephone* mutants (solid orange line) were dramatically protected. (n≥10 fish per group, 3 neuromasts per fish. Error bars: S.D.; ANOVA p value indicating significance of genotype to protection: <0.0001).

Because loss of Slc4a1b activity appears to protect *persephone* mutants from neomycin-induced hair cell loss, we hypothesized that acute pharmacological inhibition of Slc4a1b activity in wildtype fish would protect hair cells from neomycin. No specific inhibitor for Slc4a1b has been described; however, as shown above in the oocyte assay, the disulfonic stilbene derivative DIDS inhibited zSlc4a1b activity in membranes. Both DIDS and a related compound SITS have been used extensively to inhibit activity of the Slc4 family of exchangers when applied in the extracellular medium, although they also block other anion channels [30], [31]. We therefore tested if treatment with DIDS and SITS could protect hair cells from exposure to neomycin. 5 dpf wildtype zebrafish were treated for 1 hr with a range of SITS or DIDS concentrations prior to 30 min co-exposure with neomycin. After one hr recovery in fresh media, fish were euthanized, fixed and immunostained with anti-parvalbumin antibody to detect hair cells. Both DIDS and SITS significantly protect hair cells from neomycin-induced cell death (Figure 6A, 6B). All concentrations of DIDS tested showed a slight but significant protection from 200 µM neomycin (Figure 6A). The protective effect of SITS treatment showed a more marked concentration dependence, including a 3-fold increase in the number of surviving hair cells with 200 µM SITS treatment after exposure to 200 µM neomycin (Figure 6B). It is unclear why DIDS is less effective than SITS. However, its more subtle protection agrees with its effects on GTTR uptake discussed below. The results with DIDS and SITS suggest that inhibition of Slc4 type channels (including Slc4a1b) prior to and during neomycin exposure is sufficient to protect hair cells in wildtype fish.

### Bicarbonate alters susceptibility to neomycin

Because zSlc4a1b is an exchanger, we hypothesized that both chloride influx and bicarbonate efflux via zSlc4a1b are inhibited in the *persephone* mutant. The wildtype protein is predicted to efflux bicarbonate; therefore loss of Slc4a1b activity may result in elevated intracellular bicarbonate levels. To mimic this condition, we altered bicarbonate concentrations of the media and tested whether this affected survival of wildtype hair cells exposed to neomycin. We reasoned that elevated external bicarbonate would both favor import of bicarbonate via other import mechanisms, and antagonize net export of bicarbonate via Slc4a1b. In this respect, it could mimic loss of bicarbonate exchange in the *persephone* mutant. Media were made with concentrations of bicarbonate ranging from 1 to 10 times the bicarbonate concentration in standard media (0.7–7 mM) and preparations were adjusted to pH 7.2. Embryos were incubated in these media for 1 hr before addition of neomycin, and then during the 30 min neomycin exposure. Elevated bicarbonate levels significantly reduced hair cell death caused by neomycin (Figure 6C). Notably, we ran these experiments in our standard media supplemented with additional bicarbonate, which showed a pH drift of not more than 0.15 pH units per 30 min, as well as in media buffered with HEPES which prevented any pH drift during the course of the experiment (Figure S2). In both cases, hair cells showed significant protection from aminoglycosides when bicarbonate amounts were elevated in the external media. This protection showed concentration dependence. At the highest concentration (10× bicarbonate: 7 mM), we observed a 7-fold increase in hair cell survival (Figure 6C, blue line).

We next evaluated whether *persephone* mutants displayed differential sensitivity to bicarbonate exposure. We treated animals with 500 µM neomycin to avoid a ceiling effect; at lower neomycin concentrations *persephone* mutants already show significant hair cell protection. *persephone* mutants and their wildtype siblings were exposed to either 1×, 5× or 10× bicarbonate concentration (0.7, 3.5 or 7 mM) for 1 hr. 500 µM neomycin was then added for 30 min. Following rinse and recovery, individual fish were scored for hair cell survival and subsequently genotyped. Data were then parsed based on genotype. Homozygous *persephone* mutants showed a dramatic enhancement in hair cell protection mediated by elevated extracellular bicarbonate. *pers* mutants were roughly 4-fold more protected than their wildtype or heterozygous siblings (Figure 6D). Thus the loss of function mutation of Slc4a1b and application of bicarbonate show synergistic effects.

### Aminoglycoside uptake is reduced in *persephone* mutants

**Figure 7:**
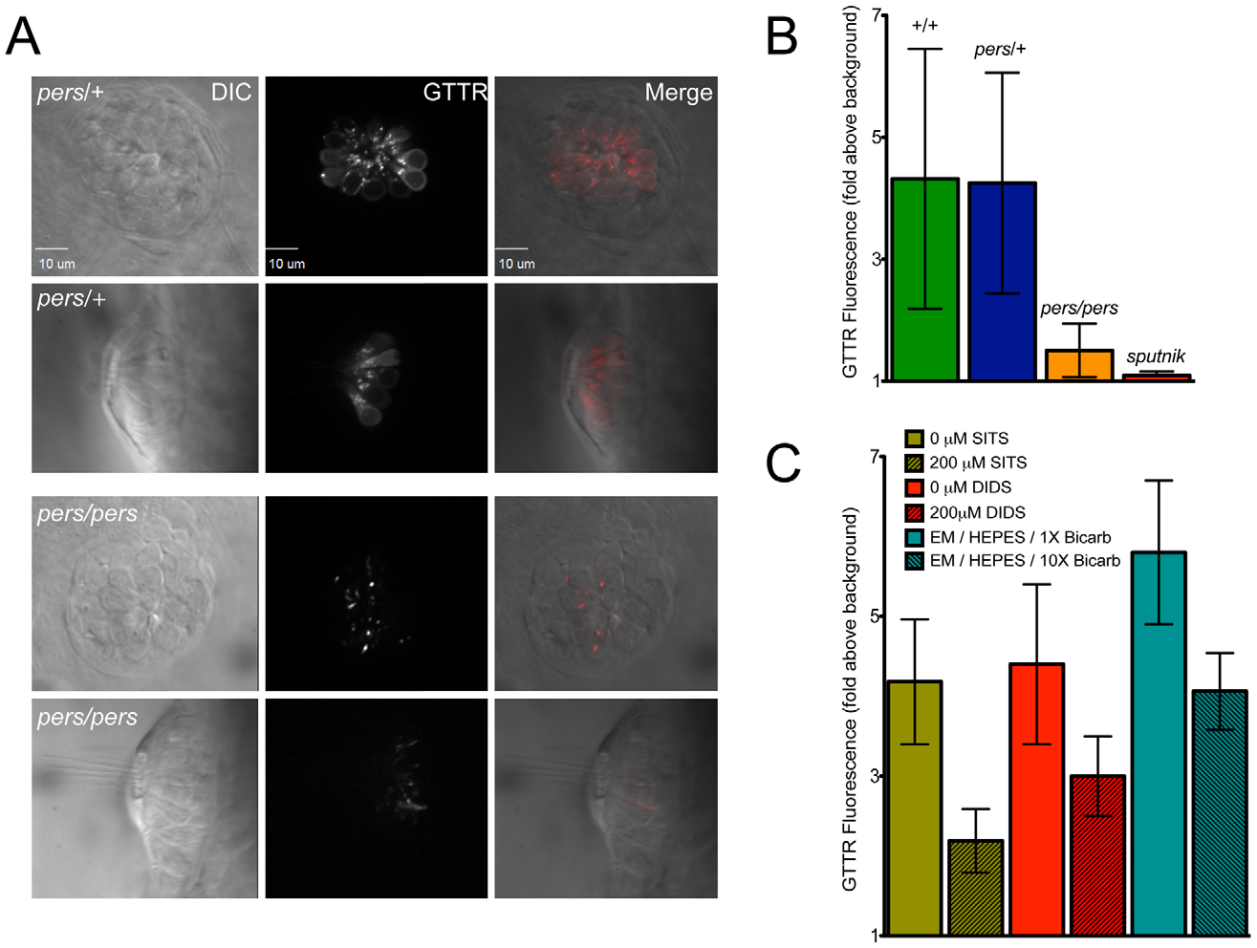
*persephone*, SLC4 inhibitors, and high bicarbonate all reduce gentamicin uptake. (A) Gentamicin-Texas Red (GTTR) labeling is shown in neuromasts in a wildtype sibling (top panels) and *persephone* mutant (bottom panels). Top down and cross sectional views are shown with identical image adjustments made to all GTTR channel images. *persephone* mutants show dramatically reduced intracellular GTTR signal. (B) Quantified GTTR signal after a 3 min exposure is reduced in *persephone* mutants compared to homozygous wildtype and heterozygous siblings. Free-swimming fish were exposed to GTTR for 3 min and rinsed by basket transfer. Neuromasts of individual fish were immediately imaged and total fluorescence signal from GTTR was quantified as described in Materials and Methods and Figure S3. After imaging, fish were individually genotyped by dCAPS. (n≥10 larvae per group, 3 neuromasts per larvae. Error bars: S.D., p value: 0.003 for difference in means between wildtype siblings and *persephone* larvae.) (C) SITS, DIDS and high bicarbonate reduce hair cell GTTR uptake. For all conditions: free-swimming wildtype 5dpf fish were treated 1 hr with either vehicle carrier, 200 µM SITS or DIDS, or 10× [bicarbonate] buffered with HEPES, and then exposed for 3 min to GTTR as in (B). GTTR uptake is significantly reduced in fish treated with concentrations of SITS, DIDS, and bicarbonate that protect hair cells from neomycin exposure. (n = 10 fish, 3 neuromasts per fish. Error bars: S.D. p value: <0.0001 for difference in means between 0 and 200 µM SITS, 0 and 200 µM DIDS, and 1× and 10× bicarbonate).

The resistance to aminoglycosides observed in the *persephone* mutant may reflect reduced uptake of aminoglycoside by *persephone* hair cells. To test this idea, we evaluated uptake of gentamicin fused to Texas Red (GTTR), used as a fluorescent indicator of uptake and trafficking of the aminoglycoside gentamicin [32], [33]. Individual 5 dpf larvae were pulse-labeled with GTTR for 3 min before being washed in standard media and imaged. GTTR labeling appeared both as diffuse signal and puncta that very specifically appear in lateral line hair cells. GTTR labeling was reduced in *persephone* mutants versus their wildtype siblings; however it still appeared in intracellular structures (Figure 7A). After collecting 3-D image stacks of GTTR pulse-labeled larvae, the fluorescent signal was quantified to determine the amount of GTTR that entered hair cells during the exposure. (See Figure S3 for an example of GTTR signal quantification.) After quantifying GTTR fluorescence in three neuromasts of 10 different fish, larvae were genotyped. Fluorescence data was then grouped by genotype. Quantified GTTR uptake differed markedly between *persephone* mutants and wildtype siblings. *pe*rs mutants showed an 85% reduction in GTTR uptake relative to their wildtype siblings (Figure 7B). This reduction was still significantly above the 97% reduction seen in the mechanotransduction mutant *sputnik* (*cdh23*), indicating a decrease, but not a block, in entry of aminoglycosides.

**Figure 8:**
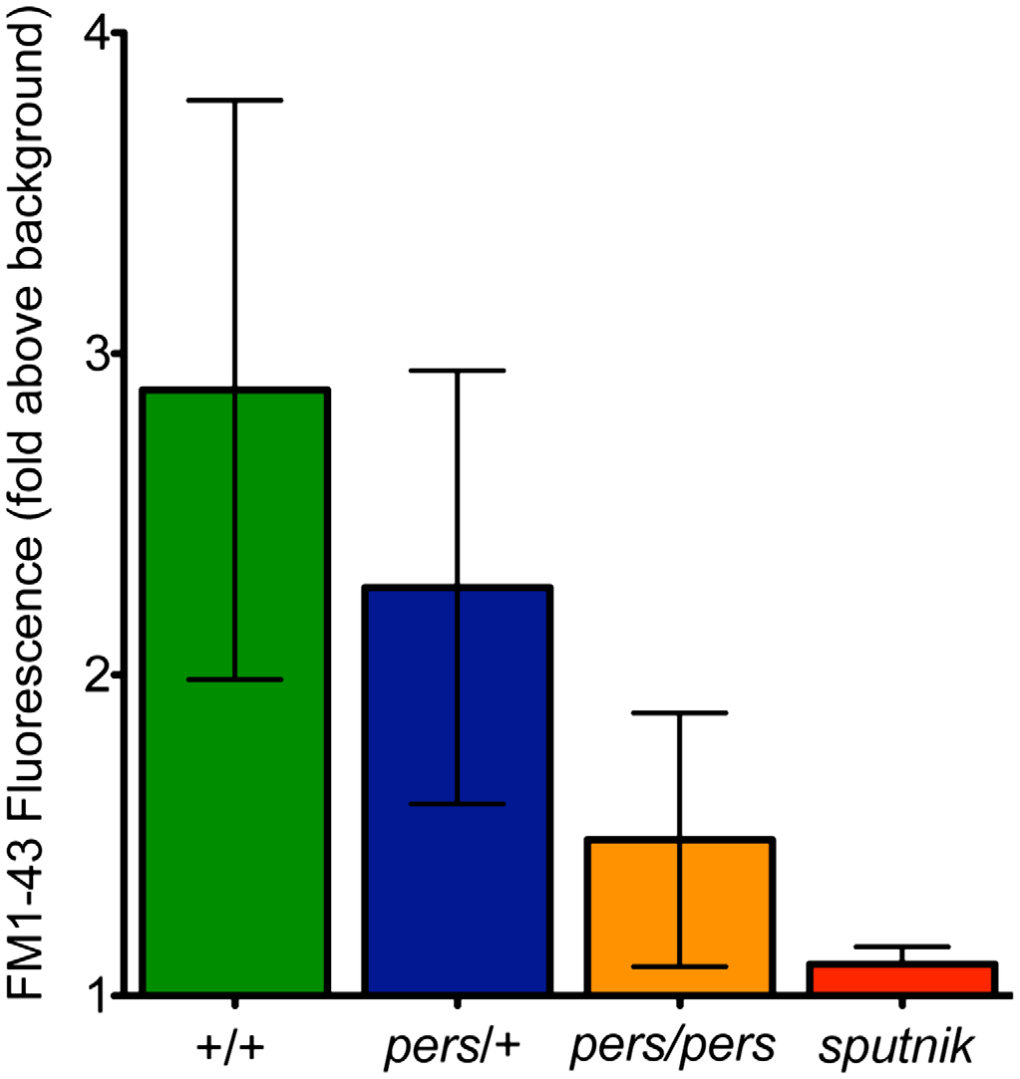
FM1-43 uptake—used as a proxy for mechanotransduction activity—is reduced in *persephone* mutants. Free swimming 5dpf progeny of an incross of *persephone* heterozygotes were individually exposed to FM1-43 for 1 min and rinsed by basket transfer. Neuromasts of individual fish were immediately imaged and total fluorescence signal from FM1-43 was quantified as described for GTTR quantification in Materials and Methods. After imaging, fish were individually genotyped by dCAPS. (n≥10 larvae per group, 3 neuromasts per larvae. Error bars: S.D., p value: 0.003 for difference in means between wildtype siblings and *persephone* larvae. +/+ and *pers*/+ larvae are not significantly different (p value 0.15). Uptake in *persephone* is significantly higher than in the loss of mechanotransduction mutant sputnik (p value 0.02.).

We next asked whether the conditions we used to mimic loss of function of zSlc4a1b-mediated exchange reduced Gentamicin-Texas Red entry into wildtype hair cells. We first evaluated GTTR uptake in wildtype larvae pretreated with either SITS or DIDS, shown above to protect hair cells from neomycin exposure. Pharmacological blockade of wildtype zSlc4a1b activity by either 200 µM SITS or 200 µM DIDS significantly reduced GTTR uptake in lateral line hair cells (Figure 8A, Green and Red columns). Notably, at concentrations of DIDS and SITS that did not protect hair cells, we saw no reduction in GTTR fluorescence (data not shown). We also asked whether high bicarbonate concentration decreased entry of Gentamicin-Texas Red into hair cells. Increasing bicarbonate in the embryo media 10-fold significantly reduced GTTR entry (Figure 8A, Teal columns). These experiments were carried out in HEPES to prevent pH drift. The fact that loss of Slc4a1b activity, pharmacological inhibition of Slc4 family mediated exchange, and altering Slc4a1b substrates all reduce aminoglycoside uptake strongly suggests that entry of aminoglycosides into hair cells can be regulated by chloride or bicarbonate ion concentrations.

Several channels have been reported to mediate aminoglycoside entry into hair cells [33], [34]. Activity of the mechanotransduction channel is clearly a key regulator of aminoglycoside entry [35]. Lateral line hair cells lacking mechanotransduction activity do not die when exposed to aminoglycosides, and do not take up labeled aminoglycosides to an appreciable degree. We asked whether mechanotransduction channel activity is altered in the *persephone* mutant. The styryl dye FM1-43 is a fluorescent molecule that intercalates into phospholipid bilayers. It specifically labels lateral line hair cells in live zebrafish larvae. Mechanotransduction mutants like sputnik (cdh23) show severely reduced FM1-43 hair cell labeling. FM1-43 can been used to block mechanotransduction in cochlear hair cells, and is a proxy for mechanotransduction activity [36], [37]. We therefore evaluated whether *persephone* mutants show reduced FM1-43 labeling relative to their wildtype siblings. A clutch from a heterozygous incross was pulse-labeled for 1 min with 0.35 mM FM1-43FX. Individual larva were washed and imaged immediately, and the FM1-43FX signal was quantified using the approach described for GTTR. Larvae were genotyped after fluorescent signal quantification, and the data were parsed by genotype. Consistent with decreased mechanotransduction activity, *persephone* mutants show a marked decrease in FM1-43 labeling (Figure 8B). The decreased uptake of both fluorescent aminoglycoside analogs and FM1-43 suggest that a fundamental effect mediating hair cell survival in *persephone* may be decreased mechanotransduction activity.

## Discussion

Although aminoglycoside antibiotics are effective against systemic infections, the associated risk of hair cell death coupled to irreversible hearing loss and vestibular deficits presents significant impediments to their clinical use. We identified *persephone* in a genetic screen based on the ability of this mutant to suppress aminoglycoside-induced lateral line hair cell loss in zebrafish larvae [9]. We mapped the genetic lesion to *slc4a1b*, which encodes a chloride/bicarbonate anion exchanger. The mutation results in a missense substitution that abolishes the chloride transport function of the exchanger. Pharmacological inhibition of the SLC4 family of proteins or exposure to increased bicarbonate (a substrate of Slc4a1b) protects hair cells of wildtype fish. In *persephone* and in these conditions that mimic the mutant phenotype, aminoglycoside entry is decreased. This reduction in aminoglycoside entry may be a consequence of decreased mechanotransduction (MET) activity; FM1-43 labeling, used as a proxy for MET activity [37], [38], is significantly decreased in *persephone*. While we cannot rule out that the inhibitors of SLC4 activity (DIDS and SITS) may have interactions with other channels, our data supports that there are likely anion gradients regulated by Slc4a1b, which in turn regulate aminoglycoside entry into lateral line hair cells. Furthermore, this regulation may act through effects on the mechanotransduction channel.

Zebrafish Slc4a1b is a member of the Slc4 family of anion transporters that are ubiquitous among eukaryotes, and expressed in occasional marine bacteria [21], [39]. Zebrafish Slc4a1b is most closely related to the Na^+^ independent, electroneutral chloride/bicarbonate exchangers SLC4A1, SLC4A2, and SLC4A3 which regulate intracellular bicarbonate and chloride for pH_i_ buffering, osmotic control and regulation of cell size [25], [40]. Cell-specific functions of SLC4A1 include distal urinary acidification and bicarbonate reabsorption by renal collecting duct Type A intercalated cells [26]. In gastric parietal cells, SLC4A2 promotes gastric acid secretion through basolateral chloride influx and bicarbonate efflux [41]. In neurons, SLC4A3 is reported to influence electrochemical potential via chloride exchange, in turn affecting neuronal excitability [42]–[45]. Mutations in the SLC4 class of exchangers are associated with several human diseases: hereditary spherocytic anemia, hereditary overhydrated stomatocytic anemia, and distal renal tubular acidosis (dRTA) [28], [46]. Although sensorineural hearing has been observed in patients with dRTA, it is most often seen in patients with mutations in other genes [47].

Several studies report SLC4 family protein expression in the mammalian cochlea [48]. SLC4A2 (AE2) has been observed in the guinea pig inner ear, and may play a role in maintenance of endolymphatic pH [48]. AE2 may also participate in regulating outer hair cell rigidity. However, this is likely via its function linking plasma membrane and cytoskeletal elements, rather than its anion exchange activity [49]. In the zebrafish, Slc4a1b is strongly expressed in ionocytes of the skin that regulate salt and pH balance, in particular in the HR (H^+^ ATPase-rich) ionocyte subtype [21]. These ionocytes maintain pH homeostasis by secreting acid from the surface of the fish [50]. While *slc4a1b* shows broad expression, its locus of action relative to hair cell function remains to be determined.

Because the reported activities of Slc4a1b may broadly affect physiology and intracellular processes, it is unclear at this time how exactly loss of its activity protects lateral line hair cells from aminoglycoside exposure. It is reasonable to assume that *persephone* mutants have altered intracellular conditions in lateral line hair cells that decrease aminoglycoside toxicity. Previous studies have shown that protonation of aminoglycoside amine groups is critical for aminoglycoside binding to ribosomal RNA and other macromolecular targets [51], [52]. However, the pKa of gentamicin is 8.2. Without severely affecting intracellular pH, it is unlikely this mechanism is the major route for protection in *persephone*. Altered intracellular pH can also affect the function of mitochondria, a locus of aminoglycoside toxicity [24], [53], [54]. However, *persephone* mutants do not show overt changes in the morphology of mitochondria or other intracellular structures in the absence of aminoglycosides. Micrographs of untreated *persephone* larvae are indistinguishable from micrographs of their wildtype siblings. Additionally, 5 dpf *persephone* mutants are morphologically and behaviorally indistinguishable from their wildtype siblings. These observations suggest that protection in *persephone* likely occurs through a hair cell specific effect rather than a broad physiological or developmental effect.

An alternative way to decrease toxicity of aminoglycosides is to alter their loading into hair cells. MET channels in the stereocilia facilitate aminoglycoside entry into hair cells, either directly or indirectly [55]. Mutations and small molecules that decrease activity of the mechanotransduction channels in hair cells both inhibit aminoglycoside entry and decrease aminoglycoside-induced hair cell death [20], [35], [36]. In the *persephone* mutant, uptake of the fluorescent aminoglycoside analog Gentamicin-Texas Red into hair cells is reduced. It is very unlikely this is due to changes in movement of aminoglycosides through the mutated Slc4a1b channel itself. Both the charge characteristics and size of SLC4 substrates preclude aminoglycosides as substrates of the exchanger [39]. It is much more likely that intracellular conditions influenced by Slc4a1b activity affect entry of aminoglycosides into the lateral line hair cells. Notably, hair cell loading of the styryl dye FM1-43, used as a proxy for mechanotranduction in zebrafish lateral line hair cells, is significantly reduced in *persephone* hair cells; however, FM1-43 labeling in *persephone* is reduced less than in mutants with no mechanotransduction—e.g. *cadherin23* [36], *myosin 6b* [56] and *protocadherin 15a* [57]. *persephone* larvae also do not exhibit behavioral phenotypes seen in mutants that have lost mechanotransduction—e.g. reduced startle behavior or minimal spontaneous swimming activity. It appears instead that *persephone* has compromised activity that is sufficient to reduce aminoglycoside (and FM1-43) uptake but not abolish all signaling from the lateral line system.

We do not at this time know how Slc4a1b activity affects lateral line hair cells. We favor the idea that compromised anion exchange activity of Slc4a1b perturbs the ionic microenvironment of the MET channel and thereby impacts aminoglycoside entry into hair cells. Previous reports indicate SLC4 proteins may assist in regulating endolymphatic potential in the mammalian cochlea [48]. Slc4a1b may similarly affect the composition of the lateral line hair cell environment. Although these hair cells are superficial, they maintain a unique apical microenvironment—the cupula–a gelatinous matrix covering the stereocilia and kinocilia [58]. Both the cupula and endolymph maintain high concentrations of potassium and chloride ions [59], [60]. pH regulation might influence endolymph potassium grandients, and aminoglycoside entry into mammalian hair cells is sensitive to ion gradients [49], [61]. Slc4a1b-mediated exchange of bicarbonate and chloride therefore may be important for maintaining local ionic conditions that enable mechanotransduction channel activity. Perturbation of the cupula environment is hypothesized to reduce the ability of aminoglycosides to either transit the MET channel directly or transit MET-dependent pathways [61].

From a clinical standpoint, transiently altering endolymph ion composition may offer a way to protect hair cells from aminoglycoside exposure, if the ionic balance can later be restored. In mammals, aminoglycosides appear to move from the stria vascularis into the endolymph and finally through the apical domain of hair cells [62]. The ionic composition of the endolymph is therefore plausibly a key regulator of aminoglycoside entry into hair cells. The *persephone* mutant indicates that zebrafish may provide a useful tool to look for manipulations of the hair cell ionic environment that decrease hair cell activity and aminoglycoside toxicity.

## Materials and Methods

### Fish

Adult *Danio rerio* (zebrafish) were maintained in the University of Washington zebrafish facility. The *persephone* mutant stock was maintained as heterozygotes on the *AB wildtype background by successive backcrossing. *persephone* mutant and wildtype (*pers/+* and *+/+*) larvae were produced from paired matings of *pers* heterozygotes. Experiments were carried out on larvae raised to 5 days post-fertilization (dpf) in embryo media (14.97 mM NaCl, 500 µM KCl, 42 µM Na_2_HPO_4_, 150 µM KH_2_PO_4_, 1 mM CaCl, 1 mM MgSO_4_, 0.714 mM NaHCO_3_, pH 7.2) unless otherwise indicated. All animal work was conducted according to relevant national and international guidelines with approval of the University of Washington IACUC.

### Mapping

Heterozygous mutant carriers were outcrossed to the polymorphic WIK wildtype zebrafish strain for mapping. Hybrid *AB/WIK carriers were identified phenotypically and intercrossed to produce progeny for linkage marker analysis. 5 dpf zebrafish were exposed to neomycin as described for the initial screen [9]. A bimodal response to neomycin was observed. To ensure accurate phenotyping after exposure to 200 µM neomycin, we designated individuals with hair cell retention greater or equal to the midpoint of the second peak of the bimodal distribution as mutants (typically ∼75% hair cell retention or better). We designated individuals with hair cell retention less than or equal to the midpoint of the lower peak of the bimodal distribution as wildtype (typically ∼5% hair cell retention or less). For bulk segregant analysis, two pools of mutant and two pools of wildtype DNAs were composed. Each pool included DNAs from 20 individuals. Distribution of markers was compared to DNA from fin clips of *AB/WIK parents and founder grandparents. Microsatellite markers for each chromosome were amplified by PCR and evaluated for cosegregation with mutant phenotypes. Linked markers were further evaluated with individual DNAs from mutant fish and wildtype fish (including both heterozygous and homozygous wildtype siblings).

For fine mapping, the following PCR primers were developed to several single nucleotide polymorphisms (SNPs) found in the course of sequencing neighboring genes. snp 1: (ndufa)

F: ATGCTTGCAACCGTGATGAAAC

R: AAATGTCCTCAACATTCCTTCGC

snp 2: (ngfr 3′)

F: GAGCACACCAAAATTAATACCTGTTAA

R: GATGTTTTTATGCTTTCCCTTGTTC

snp 3: (ngfr 5′)

F: TCTTCTGATGTTTGCGATGATGAG

R: TCTGGTTCAGTGAGTTTCTGTGGA

snp 4: (ataxin 5′)

F: GCATGACAAAATGAGGAAGTGAGG

R: CCACATTCAGCGTTCAATCTCTT

snp 5: (fb57a07 int3/4)

F: TGTTGGCCTCAATCTCTGTATCAGT

R: AACCAGAGAGTAACTCTACCTGCACA

### Genotyping

Primers were designed for derived Cleaved Amplified Polymorphic Sequence analysis (dCAPS) to enable PCR-based identification of heterozygous and mutant forms of *slc4a1b* [63]. The following PCR primers introduce an Xmn1 restriction enzyme site in an amplified fragment only if the S298F mutation is present:

F: GCT GAT AAG GTG GAT AAC ATG ATG GGA GTG TCG GAG CTG AAG ATC T

R: CAC CAG CCA AAT GCC CAC CCA CAC TCG GCC TAC GAT GTA CTC

The wildtype fragment is 213 bp. Digestion with XmnI reduces the mutant fragment size from 213 bp to 170 bp while wildtype DNA remains uncut. Bands were visualized on 2% Nusieve gels run at 90 volts for 1.5 hr.

### Drug treatments

For handling and rapid transfer, zebrafish were place in tissue culture baskets submerged in 6-well tissue culture plates containing 6 mL of media or drug solutions. Neomycin (Sigma-Aldrich N1142), gentamicin (Sigma-Aldrich G1397), kanamycin (Sigma-Aldrich K1876), and cisplatin (Sigma-Aldrich P4394) solutions were diluted in embryo medium to the indicated concentrations. Animals were treated with or without drugs for 30 min (neomycin), 6 hr (gentamicin), 24 hrs (kanamycin), or 24 hrs (cisplatin), then washed by basket transfer four times in fresh embryo medium. For neomycin treatment, fish were allowed to recover for one hr in embryo media before assessing hair cell death.

4-acetamido-4′-isothiocyanatostilbene-2,2′-disulfonic acid (SITS; Sigma-Aldrich A0554) and 4,4′-diisothiocyano-2,2′-stilbenedisulfonic acid (DIDS; Sigma-Aldrich S347523) were freshly dissolved in DMSO to stock concentrations of 1 mM. Appropriate volumes of SITS or DIDS were added to embryo medium to the indicated concentrations with final DMSO concentrations ≤0.05% in both experimental and control incubations. Fish were incubated 2 hrs in either SITS or DIDS, in the absence or presence of 200 µM neomycin during the second hr. Larvae were rinsed in standard embryo media three times, allowed to recover for 1 hr in fresh embryo media, then euthanized, fixed and immunostained with anti-parvalbumin antibody as described below. Bicarbonate media formulations were made by adjusting the amount of bicarbonate used in the standard media formulation (normally 0.7 mM). Solutions were subsequently adjusted to pH 7.2 with HCl/NaOH. Fish were maintained in embryo media formulations of indicated bicarbonate concentrations for 1 hr prior to addition of neomycin to a final concentration of 200 µM, and were then incubated for an additional 30 min. Fish were rinsed in standard embryo media four times, allowed to recover for 1 hr in the final rinse of embryo media, and assayed for hair cell survival by DASPEI staining as described below.

### Hair cell labeling

#### DASPEI

To evaluate hair cells in vivo, zebrafish were labeled with 0.005% solution of the vital dye DASPEI, (2-N-ethylpyridinium iodide, Invitrogen Molecular Probes) for 15 min as previously described [16], then rinsed twice in fresh embryo media, and anesthetized with 0.0025% MS222 (3-aminobenzoic acid ethyl ester, methanesulfonate salt, Sigma-Aldrich). Anesthetized fish were transferred with wide-bore pipettes to wide depression slides and examined on an upright fluorescent dissection scope with a DASPEI filter. 10 neuromasts of the anterior lateral line (SO1-2, IO1-4, M2, MI1-2 and O2) were examined in each fish. Each neuromast was scored 2 (wildtype bright staining, normal neuromast morphology), 1 (reduced staining/altered morphology) or 0 (little or no staining) for a total score per animal of 0–20. For a given treatment/dose, 8–12 fish (80–120 neuromasts) were evaluated. Scores were normalized as a percentage of mock-treated wildtype controls.

#### Immunofluorescence microscopy

To count hair cells directly, animals were labeled with a hair cell-specific antibody (anti-parvalbumin; Millipore). Collected embryos were euthanized and fixed overnight in 4% paraformaldehyde/PBS at 4°C. Fixed embryos were washed in PBS/0.1% Tween20 (PBST), blocked with 2% goat serum in 1× PBS, .2% Triton, 1% DMSO, .02% sodium azide, and 2 mg/ml BSA), incubated overnight at 4°C in 1∶500 mouse anti-parvalbumin antibody, washed again 4 times, and then incubated overnight at 4°C with 1∶1500 goat anti-mouse secondary conjugated to Alexa-568 (Invitrogen). Stained embryos were rinsed in PBST, and stored in 50% glycerol/PBS/sodium azide for imaging. Hair cells were counted in 5 neuromasts of the anterior lateral line (IO4, M2, MI1, MI2 and O2).

### mRNA expressionc

DNAs encoding Slc4a1b were reverse-transcribed from both wildtype and mutant fish using RNA from embryos at 5 dpf. For expression in zebrafish embryos, coding sequences for wildtype and mutant *slc4a1b* were moved into the Gateway shuttle cloning system by PCR to generate slc4a1b wildtype and mutant middle entry clones (pME-slc4a1b and pME-slc4a1b(S298F). The Green Fluorescent Protein (GFP) coding sequence was added to the 3′ end of the *slc4a1b* wildtype and mutant coding sequence by Gateway recombination using vectors p5E-CMV, pME-slc4a1b or pME-slc4a1b(S298F), p3E-GFP-PolyA, and pDEST in 3-way LR recombinase reactions. For expression in *Xenopus laevis* oocytes, *slc4a1b* and *slc4a1b-GFP* wildtype and mutant forms were subcloned into the pXT7 mRNA expression construct by PCR based cloning using primers PXT7FwdSLC4EcoRI and either PXT7RevSlc4BstEII (untagged version) or PXT7REVSLC4GFPBstEII (GFP-tagged version). Linearized pXT7 plasmids encoding transporters were transcribed with SP6 RNA polymerase using mMessageMachine (Ambion).

### Primer sequences

PXT7FwdSLC4EcoRI:

GTACCGAATTCGGTGTGATGATGCTGGACAGAGAGGAGAAGACGCTCTCC

PXT7RevSlc4BstEII:

GAGCTTATAGGTTACCTTACAGTGGCATCTGAGTTTCAGAATACAC

PXT7REVSLC4GFPBstEII:

GAGCTTATAGGTTACCTTAGTACAGCTCGTCCATGCCGAGAGTGATCC

### Transmission electron microscopy

For ultrastructural analysis, zebrafish larvae were treated for 1 hr with neomycin (50 or 200 µM) or mock-treated, and then euthanized and fixed in ice cold 4% paraformaldehyde. The caudal half of each fish was excised and used for DNA isolation and rostral half of fish was fixed in gluteraldehyde in 0.1 M sodium cacodylate +0.001% CaCl_2_ (pH 7.4, 583 mOsm) for 1 hr at 25°C with gentle agitation, and then with fresh fixative overnight at 4°C. Separate groups of larvae from the same clutch were assessed by *in vivo* DASPEI labeling of hair cells to verify efficacy of the neomycin treatment. After fixation, samples were washed three times for 10 min with 0.1 M sodium cacodylate (pH 7.4)+0.001% CaCl_2_, post-fixed in 1% osmium tetroxide in 0.1 M sodium cacodylate (pH 7.4)+0.001% CaCl_2_ for 30 min on ice in the dark, with agitation, and then washed three times for 10 min with 0.1 M sodium cacodylate (pH 7.4)+0.001% CaCl_2_. Samples were dehydrated in a graded ethanol series (10 min each in 35% EtOH, 70% EtOH, 95% EtOH, and twice in 100% EtOH), washed twice for 10 min in propylene oxide, and once for one hr each in 1∶2 and then 2∶1 mixtures of propylene oxide: Eponate resin (Electron Microscopy Sciences, Ft. Washington, PA, made per manufacturer's instructions). Tissue was then immersed 1 hr in 100% Eponate resin, and placed in fresh 100% Eponate resin overnight for infiltration. Fish were embedded in 100% Eponate resin in silicone rubber molds (Ted Pella, Redding, CA, catalog no. 10504) with zebrafish oriented lengthwise, rostral to caudal. Blocks were baked at 60°C for 18–24 hr. Sections were typically taken from mid-eye to just posterior to the eye to allow evaluation of several neuromasts in the same section (including neuromasts SO1, SO2, IO3, IO4, M2, MI1, O1, O2, OC1,and/or OP1 using the nomenclature of Raible and Kruse [64]. Semi-thin sections (∼2 mm) were cut and stained with 1% toluidine blue in 1% sodium borate. When a semi-thin section was observed in which neuromasts were present, ultrathin sections of ∼90 nm were collected on 200 mesh Athene thin-bar grids (Ted Pella, Redding, CA). Tissue was contrasted with 5% uranyl acetate in 50% methanol for 20 min, rinsed with 50% methanol and counterstained with 0.3% lead citrate in 0.1 N NaOH for 4 min then rinsed with distilled water. Samples were examined with a JEOL1200EXII transmission electron microscope and photographed digitally. The entire image was adjusted to optimize the contrast and brightness, and cropped. A trained microscopist (R.P.) blind to the treatment group and to genotype evaluated neuromast images. 5000× images of 2–3 neuromast per fish were evaluated for degree of hair cell damage. The severity of damage was graded as follows: 1 = normal hair cells; 2 = normal with occasional minor mitochondrial swelling; 3 = moderate damage including some nuclear condensation, single hair cells with cytolytic appearance (light cytoplasm, swollen cell), swollen mitochondria in many hair cells; 4 = hair cells present but all severely affected with large swollen mitochondria or pronounced nuclear condensation, flatter neuromast (fewer hair cells present); 5 = neuromast devoid of hair cells or few hair cells that are present exhibit severe damage (pyknosis, fused stereocilia).

### Chloride influx assay

Ovarian segments were excised from female *Xenopus laevis* anesthetized with 0.17% tricaine according to protocols approved by the Institutional Animal Care and Use Committee of Beth Israel Deaconess Medical Center (Department of Systems Biology, Harvard Medical School, MA, USA). Stage V–VI oocytes were defolliculated following overnight incubation at 19°C of ovarian fragments with 1.3 mg/ml collagenase Type A (Boehringer Mannheim, Germany) in MBS solution (containing (in mM): 85 NaCl, 1 KCl, 2.4 NaHCO_3_^−^, 0.41 CaCl_2_, 0.33 Ca(NO_3_^−^)_2_, 0.82 MgSO_4_^2−^, 10 HEPES, pH 7.40) with added 50 ng/ml gentamicin and 2.5 mM sodium pyruvate. Wildtype and mutant cRNAs encoding zSlc4a1b or the GFP fusion proteins were transcribed from linearized template (mMessage Machine, Ambion), and oocytes were injected with 50 nL of H_2_O with or without 10 ng cRNA unless otherwise indicated. Injected oocytes were then maintained for 3–4 days at 19°C in MBS with gentamicin and pyruvate. Na^36^Cl was from GE Healthcare (Piscataway, NJ, USA). 4,4′-Diisothiocyanostilbene-2,2′-disulfonic acid, disodium salt (Na-DIDS) was obtained from Calbiochem (San Diego, CA, USA).

Unidirectional ^36^Cl^−^ influx studies were carried out in 150 mL of solution containing (in mM) 96 NaCl, 2 KCl, 1.8 CaCl_2_, 1 MgCl_2_, 5 HEPES, pH 7.4. Total bath [Cl^−^] was 103.6 mM (0.5 µCi/well). In Cl^−^-free solutions, NaCl was replaced isosmotically with 96 mM sodium isethionate, and equimolar gluconate salts of K, Ca and Mg were substituted for the corresponding Cl^−^ salts. In some experiments, influxes were performed in the presence of DIDS. Oocytes expressing wildtype and mutant proteins were subjected to parallel measurements. Samples were counted for 3–5 min such that the magnitude of 2 SD was <5% of the sample mean.

### Slc4a1b-GFP localization

Single cell embryos were microinjected with 80 ng mRNA encoding Slc4a1b-GFP or Slc4a1b(S298F)-GFP. At 48 hpf, embryos were mounted on cover glass chambers in 1% LMP agarose/embryo media with MS222 and imaged using an inverted Marianas spinning disk system (Intelligent Imaging Innovations [3i], Denver, CO) with a Zeiss C-Apochromat 63×/1.2NA Water Objective. Images shown are maximum intensity projections from 5 slices taken at 0.33 µm steps.

### GTTR uptake

Gentamicin was conjugated to Texas Red as described in [65]. Stock gentamicin Texas Red labeled solution (GTTR) was diluted 1∶1000 into 6 mL embryo media. Individual fish (5 dpf) were transferred to GTTR in embryo media and incubated for the indicated times. Larvae were then washed 3× in embryo media, and immediately imaged using an inverted 3i Marianas spinning disk system. Larvae were anesthetized in MS222 and immobilized by placement under a weighted mesh for image stability. 3-D image stacks were collected of entire neuromasts at Z increments of 0.33 µm. Total fluorescence of neuromasts was calculated using 3i Slidebook Image Analysis software. A mask was drawn around visually identified neuromasts. An identical mask outside the neuromast was used to quantify background. Neuromast and background signals were segmented to discard points less than 2 standard deviations above the mean background value. The value of the neuromast signal was then divided by the background signal to yield the relative intensity of the neuromast signal relative to background.

### Statistics

Graphpad Prism (GraphPad Software) was used for statistical analyses. To evaluate whether mutant and wildtype larvae showed differential response to a concentration range of a drug or treatment, two-way ANOVA tests were performed. To evaluate whether genetic background or drug treatment affected GTTR uptake, t-tests were performed.

## Supporting Information

Figure S1 Alignment of zslc4a1b homologs shows evolutionary conservation of Ser 298 mutated in the *persephone*. The serine residue is invariant across a diverse range of taxa.
(TIF)

Figure S2 Bicarbonate-mediated hair cell protection in EM buffered with HEPES. 5 day old zebrafish were exposed for 1 hour to embryo media with either the standard amount of bicarbonate (0.714 mM) or 10 fold this amount (0.7 mM). Both solutions were held at pH 7.2 by addition of 10 mM HEPES. Neomycin was added at the indicated concentrations for 30 minutes. Larvae were rinsed three times in fresh media, and allowed to recover for 1 hour. Hair cell survival was assessed by hair cell counts. (n≥10 fish per group, 3 neuromasts per fish. Error bars: S.D.; ANOVA p value indicating significance of bicarbonate to protection: <0.001).
(TIF)

Figure S3 Quantification of GTTR signal. To quantify GTTR signal, 3-D image stacks were collected of entire neuromasts at z-increments of 0.4 µm. Total fluorescence of neuromasts was calculated in individual neuromasts using 3i Slidebook Image Analysis software. A mask was drawn around visually identified neuromasts. The image on the top left shows a mask outlining a neuromast. Pixels within this mask are shown in blue; GTTR signal is red. A second identical mask was placed outside the neuromast to quantify background. Neuromast and background signals were segmented to discard points less than 2 standard deviations above the mean background value. The pixels that meet this criteria are shown in blue in the top right image. These two masks were then combined to generate a mask (the signal mask) that contained the region of the neuromast and required that values in that the mask be at least the mean value of the background plus two standard deviations. The bottom panels show this mask at different z-planes for the neuromast shown in the top panel. Blue points represent those points included in the mask. We calculated the mean intensity of the blue points in the signal mask. This mean value of the neuromast signal was then divided by the mean background signal to yield the relative intensity of the neuromast signal relative to background.
(TIF)
