# Supplementary figures and images for: Loss of Slc4a1b Chloride/Bicarbonate Exchanger Function Protects Mechanosensory Hair Cells from Aminoglycoside Damage in the Zebrafish Mutant persephone
Source: PLoS Genet. 2012 Oct 11;8(10):e1002971. doi: 10.1371/journal.pgen.1002971 (PMC3469417; doi:10.1371/journal.pgen.1002971)

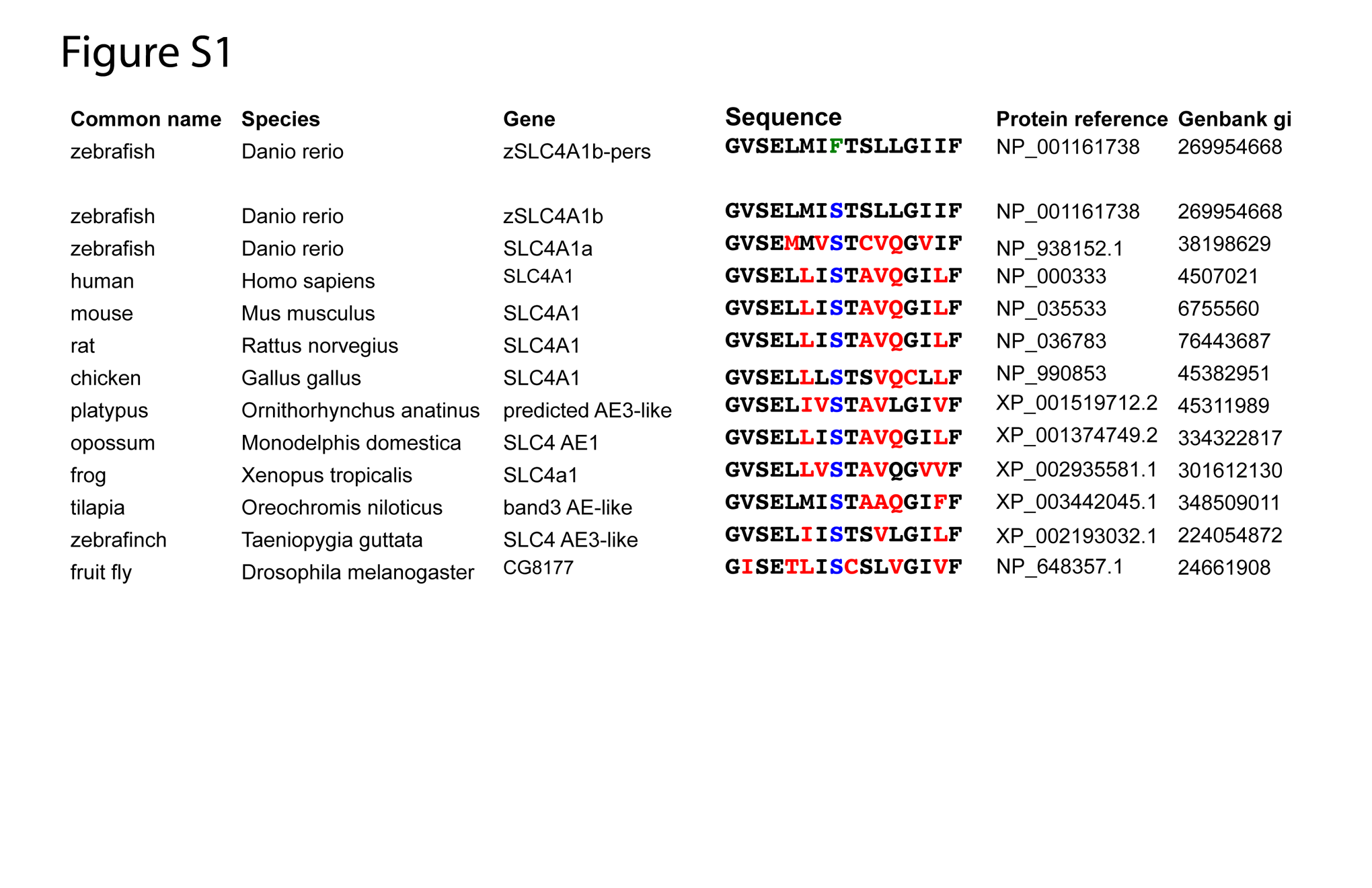

Supplement: Figure S1 — Alignment of zslc4a1b homologs shows evolutionary conservation of Ser 298 mutated in the persephone. The serine residue is invariant across a diverse range of taxa. (TIF) [file pgen.1002971.s001.tif]

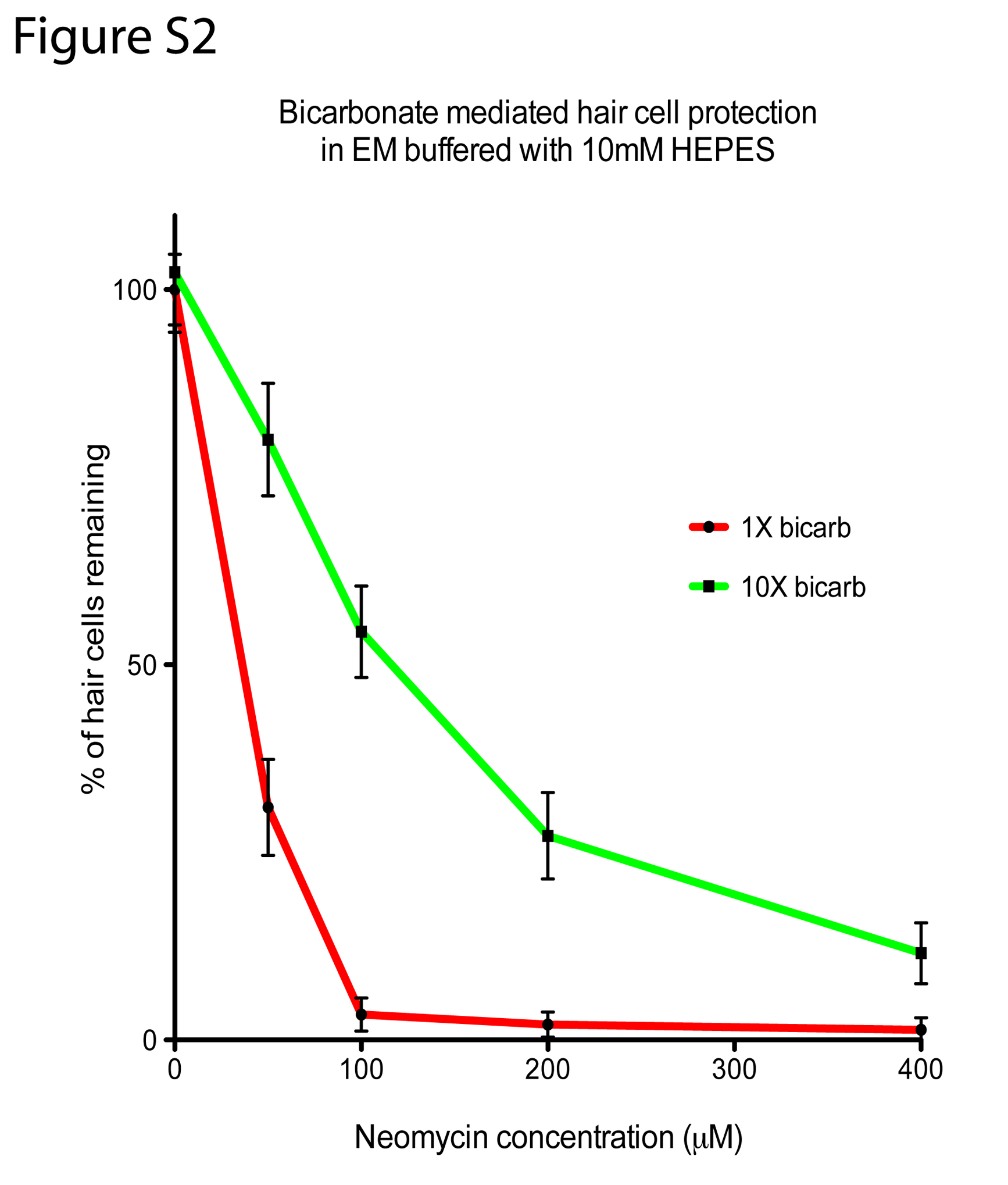

Supplement: Figure S2 — Bicarbonate-mediated hair cell protection in EM buffered with HEPES. 5 day old zebrafish were exposed for 1 hour to embryo media with either the standard amount of bicarbonate (0.714 mM) or 10 fold this amount (0.7 mM). Both solutions were held at pH 7.2 by addition of 10 mM HEPES. Neomycin was added at the indicated concentrations for 30 minutes. Larvae were rinsed three times in fresh media, and allowed to recover for 1 hour. Hair cell survival was assessed by hair cell counts. (n≥10 fish per group, 3 neuromasts per fish. Error bars: S.D.; ANOVA p value indicating significance of bicarbonate to protection: <0.001). (TIF) [file pgen.1002971.s002.tif]

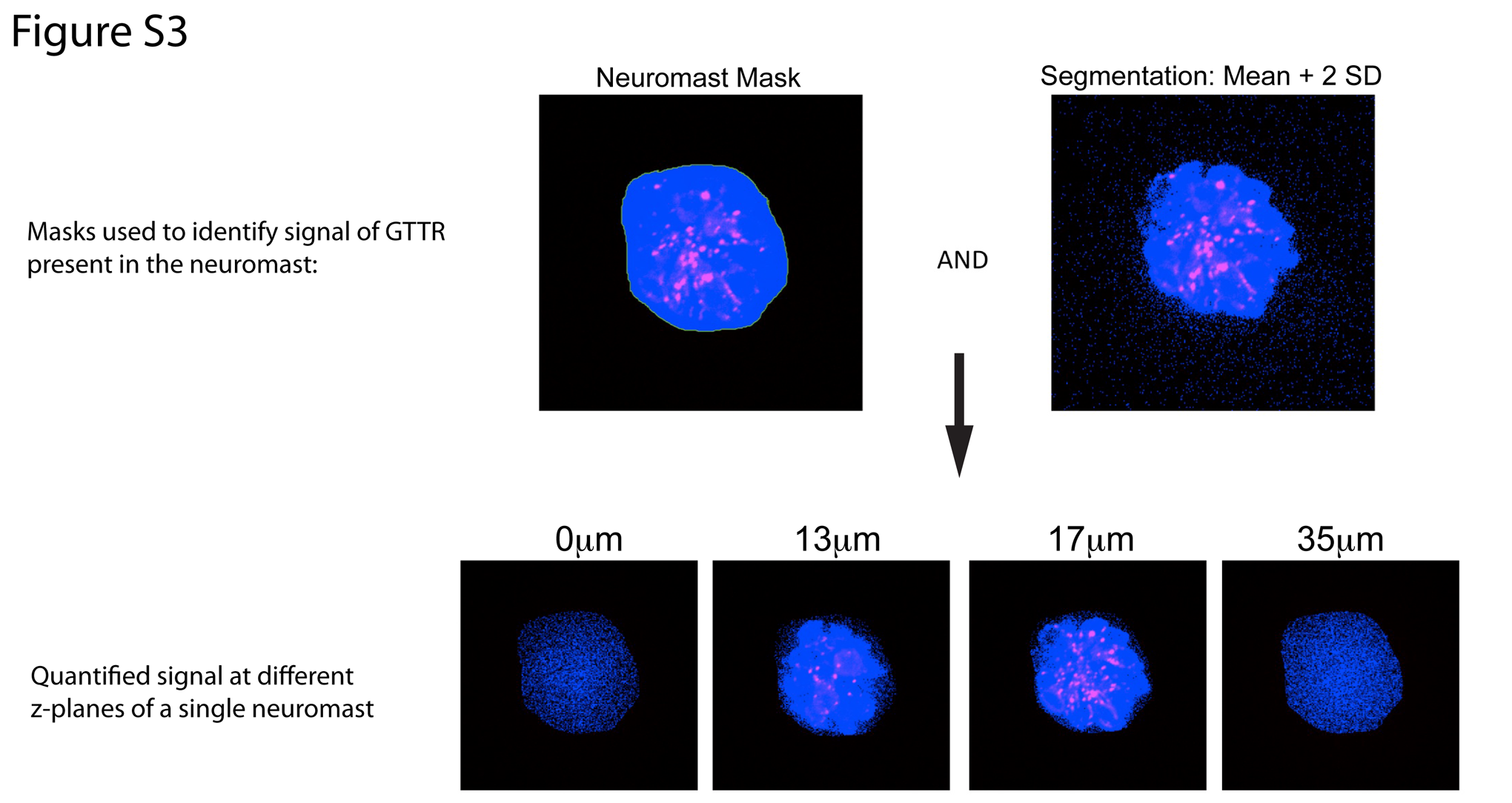

Supplement: Figure S3 — Quantification of GTTR signal. To quantify GTTR signal, 3-D image stacks were collected of entire neuromasts at z-increments of 0.4 µm. Total fluorescence of neuromasts was calculated in individual neuromasts using 3i Slidebook Image Analysis software. A mask was drawn around visually identified neuromasts. The image on the top left shows a mask outlining a neuromast. Pixels within this mask are shown in blue; GTTR signal is red. A second identical mask was placed outside the neuromast to quantify background. Neuromast and background signals were segmented to discard points less than 2 standard deviations above the mean background value. The pixels that meet this criteria are shown in blue in the top right image. These two masks were then combined to generate a mask (the signal mask) that contained the region of the neuromast and required that values in that the mask be at least the mean value of the background plus two standard deviations. The bottom panels show this mask at different z-planes for the neuromast shown in the top panel. Blue points represent those points included in the mask. We calculated the mean intensity of the blue points in the signal mask. This mean value of the neuromast signal was then divided by the mean background signal to yield the relative intensity of the neuromast signal relative to background. (TIF) [file pgen.1002971.s003.tif]
